# Transcriptome analysis of pancreatic cells across distant species highlights novel important regulator genes

**DOI:** 10.1186/s12915-017-0362-x

**Published:** 2017-03-21

**Authors:** Estefania Tarifeño-Saldivia, Arnaud Lavergne, Alice Bernard, Keerthana Padamata, David Bergemann, Marianne L. Voz, Isabelle Manfroid, Bernard Peers

**Affiliations:** Laboratory of Zebrafish Development and Disease Models (ZDDM), GIGA, University of Liège, Avenue de l’Hôpital 1, B34, 4000 Sart Tilman, Liege, Belgium

**Keywords:** RNA-seq, Comparative transcriptomics, Pancreas, Endocrine cells, Acinar cells, Ductal cells

## Abstract

**Background:**

Defining the transcriptome and the genetic pathways of pancreatic cells is of great interest for elucidating the molecular attributes of pancreas disorders such as diabetes and cancer. As the function of the different pancreatic cell types has been maintained during vertebrate evolution, the comparison of their transcriptomes across distant vertebrate species is a means to pinpoint genes under strong evolutionary constraints due to their crucial function, which have therefore preserved their selective expression in these pancreatic cell types.

**Results:**

In this study, RNA-sequencing was performed on pancreatic alpha, beta, and delta endocrine cells as well as the acinar and ductal exocrine cells isolated from adult zebrafish transgenic lines. Comparison of these transcriptomes identified many novel markers, including transcription factors and signaling pathway components, specific for each cell type. By performing interspecies comparisons, we identified hundreds of genes with conserved enriched expression in endocrine and exocrine cells among human, mouse, and zebrafish. This list includes many genes known as crucial for pancreatic cell formation or function, but also pinpoints many factors whose pancreatic function is still unknown. A large set of endocrine-enriched genes can already be detected at early developmental stages as revealed by the transcriptomic profiling of embryonic endocrine cells, indicating a potential role in cell differentiation. The actual involvement of conserved endocrine genes in pancreatic cell differentiation was demonstrated in zebrafish for *myt1b*, whose invalidation leads to a reduction of alpha cells, and for *cdx4*, selectively expressed in endocrine delta cells and crucial for their specification. Intriguingly, comparison of the endocrine alpha and beta cell subtypes from human, mouse, and zebrafish reveals a much lower conservation of the transcriptomic signatures for these two endocrine cell subtypes compared to the signatures of pan-endocrine and exocrine cells. These data suggest that the identity of the alpha and beta cells relies on a few key factors, corroborating numerous examples of inter-conversion between these two endocrine cell subtypes.

**Conclusion:**

This study highlights both evolutionary conserved and species-specific features that will help to unveil universal and fundamental regulatory pathways as well as pathways specific to human and laboratory animal models such as mouse and zebrafish.

**Electronic supplementary material:**

The online version of this article (doi:10.1186/s12915-017-0362-x) contains supplementary material, which is available to authorized users.

## Background

Pancreas is a vital organ playing crucial function in the metabolism of all vertebrates. Acinar cells, the most abundant cell type of the pancreas, produce the digestive enzymes that are conveyed to the gut by the pancreatic ducts. The pancreatic endocrine cells are grouped in the Langerhans islets and secrete diverse hormones controlling metabolism and glucose homeostasis. Five endocrine cell subtypes (alpha, beta, delta, PP, and epsilon cells) have been described in pancreatic islets, each characterized by the expression of a particular hormone (glucagon, insulin, somatostatin, pancreatic polypeptide, and ghrelin, respectively). Many transcriptomic studies have been focused on pancreatic endocrine cells, and notably on beta cells, due to their implication in the development of diabetes. Microarrays and RNA sequencing (RNA-seq) were conducted on endocrine pancreatic cells isolated from human (healthy or diabetic persons) or rodents at adult and embryonic stages [1–9]. More recently, RNA-seq performed on endocrine cell types isolated by FACS or at single-cell level allowed to define the genes enriched in each endocrine cell type in human and mice [7, 10–21]. However, no comprehensive interspecies comparison has been performed so far to define the conserved signatures for each pancreatic cell type, except for two studies comparing human and murine beta cells and reporting some notable differences between these two mammalian species [12, 21]. Comparison of transcriptomes between species is a straightforward approach to identify tissue-specific or cell type-specific genes playing crucial functions in the physiology of the studied tissue or cell. Indeed, if a gene is essential in a differentiated cell, strong constraints will maintain its expression throughout evolution and its tissue-specific expression will be detected in most species. In accordance to this view, several studies have reported conservation of organ-specific expression for a large set of genes among species [22–24]. Nevertheless, there are also striking divergences in gene expression patterns even between close vertebrate species such as human and mice, which may contribute to physiological adaptations [25, 26]. Comparative studies between evolutionary distant species are useful to identify the set of genes displaying highly conserved cell type-specific expression and likely playing a fundamental function in the studied cells. Such analyses have not been performed yet on pancreatic cells due to the lack of transcriptomic data from pancreatic cells isolated from lower vertebrates such as zebrafish. To tackle this lack of knowledge, we first determined the transcriptomic landscape of the three major endocrine cell types (alpha, beta, and delta cells) as well as of the acinar and ductal cells from zebrafish. Analysis of these zebrafish datasets allowed us to define the signature of each cell type. Then, comparison with published human and murine pancreas data led to a definition of the conserved signatures. Furthermore, by determining the transcriptome of endocrine cells from early stage embryos, we identified genes expressed during endocrine cell differentiation and putatively involved in this process; among them, *myt1b* and *cdx4* are shown to be essential for endocrine cell differentiation in zebrafish. Thus, our list of pancreatic conserved genes represents a useful resource for studies related to pancreatic development and disease such as diabetes and pancreatic cancer.

## Results

### Transcriptomic profiles of the different pancreatic cell types isolated from adult zebrafish

We purified the different pancreatic cell types from adult zebrafish using a series of transgenic reporter lines allowing the selection of these distinct cells by fluorescence-activated cell sorting (FACS). Acinar cells were obtained from the BAC transgenic lines *Tg(ptf1a:GFP)* [27]. The endocrine beta and delta cells were isolated, respectively, from the transgenic lines *Tg(ins:GFP)* ^*ulg021Tg*^ (see Methods section) and *Tg(sst2:GFP)* [28]; the alpha cells were obtained from the *Tg(gcga:GFP)*/*Tg(ins:NTR-mCherry)* line through selection of GFP^+^/mCherry cells (as many beta cells were found to express Tg(gcga:GFP) transgene at a lower level, Additional file 1: Figure S1). RNA-seq was performed on three independent preparations for each cell type, except for acinar cells, for which four replicates were prepared. About 60 million of paired-end reads were obtained from each Illumina library, 80% of which mapped to the zebrafish genome. We previously reported the transcriptome of pancreatic ductal cells by using the same procedure on the *Tg(nkx6.1:GFP)* ^*ulg004Tg*^ transgenic line [29], and these data were compared in the present study with endocrine and acinar cell transcriptomes.

**Fig. 1:**
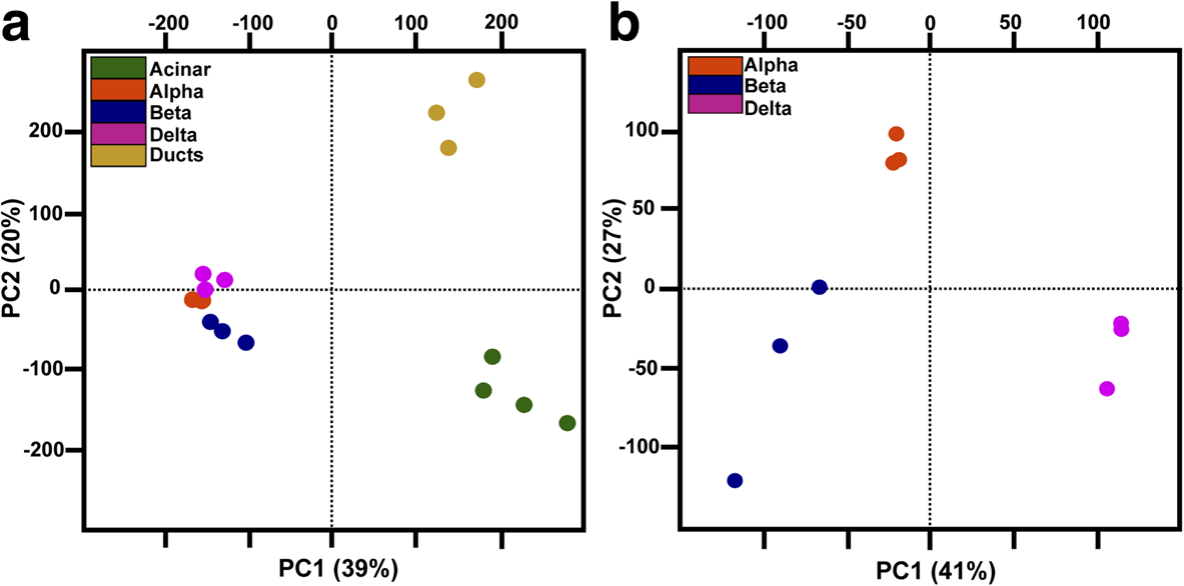
Global analysis of the zebrafish pancreatic RNA-seq data. **a** Principal component analyses (PCA) of gene VSD (Variance stabilizing transformation) calculated by DESeq package for the 16 zebrafish pancreatic datasets. **b** PCA of gene VSD for beta, alpha, and delta cells (nine samples in total). The PCA plots show a close clustering of all replicates and distinct clusters for each pancreatic cell type. PCAs were calculated using all the 33,726 genes annotated on Zv9 version 75 ensembl

Principal component analysis (PCA) of all these pancreatic RNA-seq datasets showed a tight clustering of all replicates for each pancreatic cell type (Fig. 1a), underscoring the high reproducibility of the data. As expected, the PCA also revealed a closer clustering of the three endocrine cell subtypes compared to the ductal and acinar cell types; however, when PCA is performed only with the endocrine datasets, clear distinct transcriptome profiles are observed for the alpha, beta, and delta cell subtypes (Fig. 1b). Comparison of the expression levels of various known markers of each pancreatic cell type confirmed the high purity of each cell preparation. Indeed, *glucagon a* (*gcga*), *insulin* (*ins*), and *somatostatin 2* (*sst2*) were selectively detected at very high levels in alpha, beta, and delta cell libraries, respectively, representing in average 24%, 10%, and 32% of the total reads number in the corresponding libraries, while being detected at much lower levels in the other libraries (Table 1). The *trypsin* (*try*) and *chymotrypsin-like elastase member1* (*cela1*) genes were the strongest expressed genes in acinar cells representing each about 10% of all total reads of the acinar cell libraries. These two acinar markers were not detected at significant levels in endocrine datasets consistent with an accurate cell sorting. Among the genes expressed at highest levels in ducts, we find the *cldnb*, *sdc4*, and the *epcam* genes coding for cell adhesion molecules, each representing less than 1% of total reads of ductal datasets. All these results indicate an accurate and reproducible sorting of the different pancreatic cells allowing the identification of genes selectively expressed in each pancreatic cell type. Expression values for all genes in all samples are shown in Additional file 2: Table S1 and Additional file 3: Tables S2.

**Table 1:** Percentage of the reads obtained for highest expressed markers in each type of library

Gene	Alpha	Beta	Delta	Acinar	Duct
gcga	23.8	0.5	0.9	0.0	0.0
ins	0.1	10.1	0.0	0.0	0.0
sst2	0.2	0.1	31.5	0.0	0.0
try	0.1	0.0	0.1	9.8	1.7
CELA1	0.1	0.0	0.1	12.7	1.7
cldnb	0.0	0.0	0.0	0.0	0.5
epcam	0.0	0.0	0.0	0.0	0.8
sdc4	0.0	0.0	0.0	0.0	0.5

Percentages of mapped reads obtained for some genes on the total number of mapped reads in each type of libraries (Numbers are the means of percentages obtained from replicates)

### Identification of genes enriched in endocrine, acinar, and ductal pancreatic cells

**Fig. 2:**
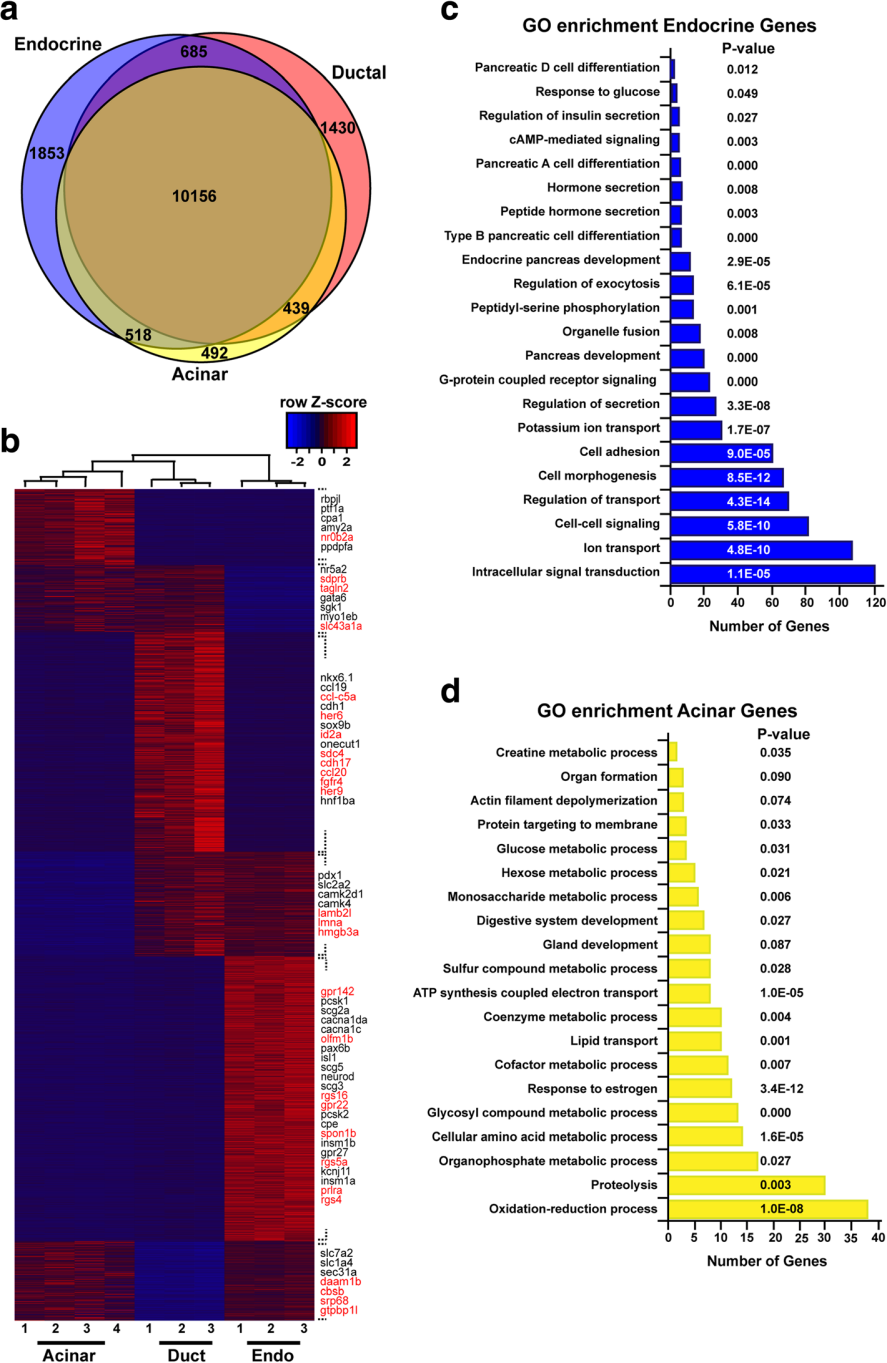
Transcriptomic signatures of zebrafish endocrine, ducta,l and acinar cells. **a** Venn diagram showing the number of genes with acinar-, endocrine- and ductal-enriched expression obtained with DESeq2 using a cut-off ratio of four-fold and adjusted *P* < 0.05. **b** Heatmap plot showing the expression pattern of all differentially expressed genes. Genes listed on the right side of the plot are examples of either known markers (black) or new genes with endocrine-, acinar-, and ductal-enriched expression discovered by this analysis (red). The three endocrine samples were generated in silico by combining the raw data obtained from alpha, beta, and delta cells as described in the Methods section. **c** and **d** Gene ontology enrichment analysis for the 1853 genes enriched in endocrine cells (**c**) and the 492 genes enriched in acinar cells (**d**) displaying the most enriched biological process

The clear distinct transcriptomic profiles observed for endocrine, acinar, and ductal cells prompt us to identify, in a first step, all genes presenting a differential expression in these three pancreatic tissues (with at least a four-fold enrichment and adjusted *P* < 0.05). By using these cut-off values, 1853, 1430, and 492 genes were found to be enriched in endocrine, ductal, and acinar cells, respectively (Fig. 2a and b, gene lists are given in Additional file 4: Table S3). As expected, the zebrafish endocrine-specific genes include several orthologs of mammalian genes known as endocrine markers such as those involved in hormone regulation and secretion, like the proprotein convertases (*pcsk1* and *pcsk2*), carboxypeptidase E (*cpe*), secretogranins (*scg2a*, *scg3*, and *scg5*), ATP-dependent potassium channels (the *kcnj11* and *Sur1*/*abcc8* subunits), and the voltage-dependent type calcium channels (*cacna1c* and *cacna1da*), among others. Moreover, most of the transcription factors previously shown to be crucial for the differentiation of endocrine cells (*neurod*, *isl1*, *pax6b*, *insm1a*, etc.) [30] are detected in the endocrine signature validating our RNA-seq data. Interestingly, many other transcription factors, whose function in endocrine cells is still not known, are present in this list such as *egr4*, *creb3l1*, *lmo1*, *cdx1b*, or *cdx4* (Fig. 2b). Gene ontology (GO) enrichment analysis using DAVID revealed known biological pathways in endocrine cells such as “potassium ion transport”, “regulation of secretion” and “regulation of exocytosis” as well as “response to glucose”, and “G-protein coupled receptor signaling” (Fig. 2c). Indeed, many G-couple protein receptors (GPCRs) and several regulators of G-protein signaling, like *gpr12*, *gpr22*, *gpr27*, or *gpr63* as well as *rgs4*, *rgs5a*, *rgs8*, or *rgs17*, among others, were found enriched in the endocrine cells. While some of these regulatory proteins have been previously reported to control pancreatic islet activity [31, 32], others were not yet known to have a selective expression in pancreatic endocrine cells nor to play a role in islet physiology.

Similar observations were done for the acinar and ductal cell transcriptomic signature. Indeed, as expected, many genes coding for digestive enzymes, such as *trypsin* (*try*), *elastase* (i.e., *ela* and *cela*), *trypsin-like* (*tryl*/*zgc:66382*), *amylase* (*amy2a*) as well as the known acinar transcription factors *ptf1a* and *rbpjl*, were found enriched in acinar cells. The acinar gene list also includes novel markers such as genes encoding for the transcription factors *esr2a*, *klf15*, or *nr0b2a* (Fig. 2b and Additional file 4: Table S3). As expected, GO enrichment analysis revealed, among the biological processes active in acinar cells, “Gland development” and “Digestive system development”, “Cellular amino acid metabolic process”, “Proteolysis” as well as “ATP synthesis coupled electron transport” (Fig. 2d). As for the duct transcriptome, the analysis reveals novel markers such as *id2a* and *frzb* in addition to known markers like *sox9b*, *nkx6.1*, *onecut1*, and *ctgfa*, as we recently described [29]. All together, these analyses confirm that many known markers and biological pathways display the same pancreatic enrichment in zebrafish as in mammals, and also highlight many novel cell type-specific genes not previously reported to display such selective expression in mammals. This led us to compare comprehensively the endocrine- and exocrine-enriched genes across zebrafish and mammalian species, thereby defining the conserved specific signatures among vertebrates.

### Comparison of the pancreatic endocrine and exocrine transcriptomic signatures across zebrafish, mouse, and human

**Fig. 3:**
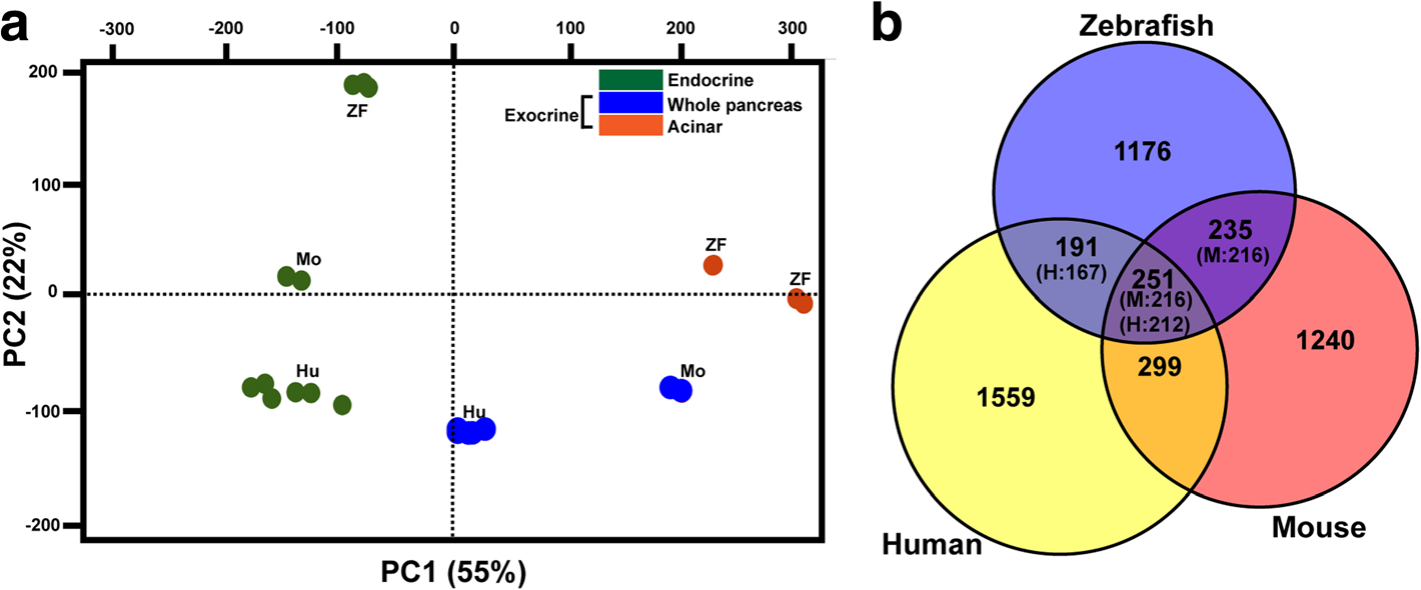
Conservation of the pancreatic endocrine signature among vertebrates. **a** Principle component analysis (PCA) performed on human and mouse whole pancreas and islet RNA-seq datasets and including the zebrafish endocrine and acinar datasets. The analysis was performed using the 9393 genes displaying 1-1-1 orthology relationship between zebrafish, mouse, and human using a total of 24 RNA-seq samples. The endocrine datasets of zebrafish, mouse, and human cluster along the PC1 axis representing 55% of the variance, indicating a conserved endocrine signature. The human pancreatic sample enriched in acinar tissue [9] clusters with the human whole pancreatic samples [37] due to the very high proportion of acinar cells in pancreas. **b** Venn diagram showing the number of endocrine-enriched genes found only in zebrafish, mouse, or human and those displaying conserved endocrine-enrichment in two species or in the three species (shown in intersections). Due to gene duplications in some species and often in zebrafish, the number of corresponding murine (M) or human (H) orthologous genes is given in brackets in each intersection. The full list of conserved endocrine-enriched genes is given in Additional file 5: Table S4

The evolutionarily conserved expression of a gene in a specific cell type is a strong argument for its crucial function in that cell. This concept is widely supported by numerous studies showing conserved expression of many transcription factors in development and cell differentiation [23, 33], including pancreatic cells [34, 35]. To perform interspecies transcriptome comparison, we retrieved, from public databases, several RNA-seq datasets obtained on murine and human pancreatic islets, whole pancreas [11, 36–38], as well as a human pancreatic sample enriched in acinar tissue [9]. While mouse RNA-seq data are not presently available for acinar enriched preparations, the genes with either endocrine-enriched or exocrine-enriched expression (named hereafter as “endocrine or exocrine genes”) can nevertheless be identified by comparing RNA-seq from purified pancreatic islets versus RNA-seq from whole pancreas (composed of more than 90% of exocrine cells). When a global comparison of the zebrafish endocrine and acinar datasets with the murine and human pancreatic data was performed using a PCA plot analysis, we observed that the data clustered according to the tissue type (endocrine and acinar) in the first component (PC1: representing 55% of variance), while the data tended to cluster according to the species in the second component (PC2: representing 22% of variance) (Fig. 3a). Thus, this global analysis suggests the existence of sets of genes displaying an evolutionary conserved endocrine and exocrine expression. Endocrine and exocrine genes were identified from human and murine datasets by selecting all genes presenting at least four-fold higher expression in each pancreatic tissue (with adjusted *P* < 0.05). Next, we compared the endocrine and exocrine gene lists between the three vertebrate species. The Venn diagram on Fig. 3b indicates that, while a large fraction of endocrine-enriched genes are species-specific, some sets of genes display a conserved endocrine-enriched expression in two species or in the three species. Indeed, among the 1853 zebrafish endocrine genes, 251 are also classified as “endocrine” in human and mice. This endocrine signature, conserved between zebrafish, mice and human (named “ZMH”), includes many factors known to be involved in hormone maturation, secretion, and regulation. Many transcription factors crucial for endocrine cell differentiation [30, 39] are found in this ZMH conserved endocrine signature (Table 2), thereby validating this method to identify genes with important physiological function. The ZMH endocrine genes (given in Additional file 5: Table S4) also comprise components of signaling pathways reported to control pancreatic endocrine cells in mammals such as the glutamate receptor *gria2a* and *gria2b* [40], regulators of G-protein signaling (*rgs4* and *rgs16*) [32, 41], *urocortin3* [5, 42], ion channels (*abcc8*, *cacna1*, and *scn1lab*), and calcium dependent proteins (*c2cd4* and *scgn*). This indicates that many regulatory processes controlling endocrine cell development and physiology have been maintained from fish to human. Interestingly, the ZMH endocrine genes include several regulatory genes whose function in pancreatic cells is still unknown or not well defined such as the kinases *map3k15* and *mast1*, the signaling factor *gpr158*, as well as the transcription factors *npas4a*, *lmo1*, *fev*, and *etv1*. Similarly, the comparison of the exocrine-enriched genes across zebrafish, mice, and human shows that a fraction of genes displays conserved exocrine enrichment (Additional file 6: Figure S2) – among the 2361 zebrafish exocrine genes (combined acinar and ductal genes from Fig. 2a), 127 show exocrine enriched expression also in human and mice. This conserved exocrine signature (Additional file 7: Table S5) includes, as expected, many digestive enzymes as well as known exocrine transcription factors such as *ptf1a*, *rbpjl*, and *nr5a2* [36, 43], but also regulatory genes with unknown pancreatic function, like *klf15*.

**Table 2:** List of conserved endocrine-enriched transcription factors

Gene	Endocrine	Acinar	Duct	Conservation
runx1t1	535	2	7	ZHM
isl1	8613	41	76	ZHM
myt1la	96	0	4	ZHM
fev	5884	128	44	ZHM
neurod1	28467	260	300	ZHM
ascl1a	86	0	1	ZHM
rfx6	1517	26	5	ZHM
pax6b	8059	47	23	ZHM
insm1b	1200	6	39	ZHM
npas4a	4497	11	114	ZHM
arxa	1697	38	1	ZHM
myt1a	532	0	17	ZHM
insm1a	3419	10	248	ZHM
lmo1	1425	4	1	ZHM
etv1	284	0	0	ZHM
esrrga	159	8	2	ZM
rfx2	1590	46	253	ZM
sim1a	164	2	1	ZM
mnx1	1368	144	3	ZM
cdx4	144	0	16	ZM
bhlhe41	2984	712	640	ZM
lmx1bb	145	2	18	ZM
znf516	219	3	31	ZM
esr1	1701	10	59	ZH
tbx2b	1246	7	8	ZH
pgr	399	4	15	ZH
nkx3.2	293	0	26	ZH
nr0b1	284	14	8	ZH
sox11b	49	1	10	ZH

Transcription factors enriched in endocrine cells in zebrafish, human and/or mouse. The level of expression in endocrine, acinar, and ductal cells is shown for each gene in the second, third, and fourth column, respectively. The degree of expression pattern conservation is shown in the last column as ZHM: endocrine-enriched in zebrafish, human, and mouse; ZM: endocrine-enriched in zebrafish and mouse; ZH: endocrine-enriched in zebrafish and human

The interspecies comparison of both exocrine and endocrine signature also indicates that many genes display the same enrichment in only two species (e.g., “HM”: between human and mice; “ZM”: zebrafish and mouse). As expected, the number of conserved HM endocrine and exocrine genes is higher than the number of conserved ZH and ZM genes. Overall, this global analysis indicates that, while significant divergence is observed between species at the level of endocrine and exocrine signatures, hundreds of genes have nevertheless maintained a common expression pattern, among which are most of the known pancreatic regulatory genes.

### Identification of genes expressed in embryonic endocrine cells

**Fig. 4:**
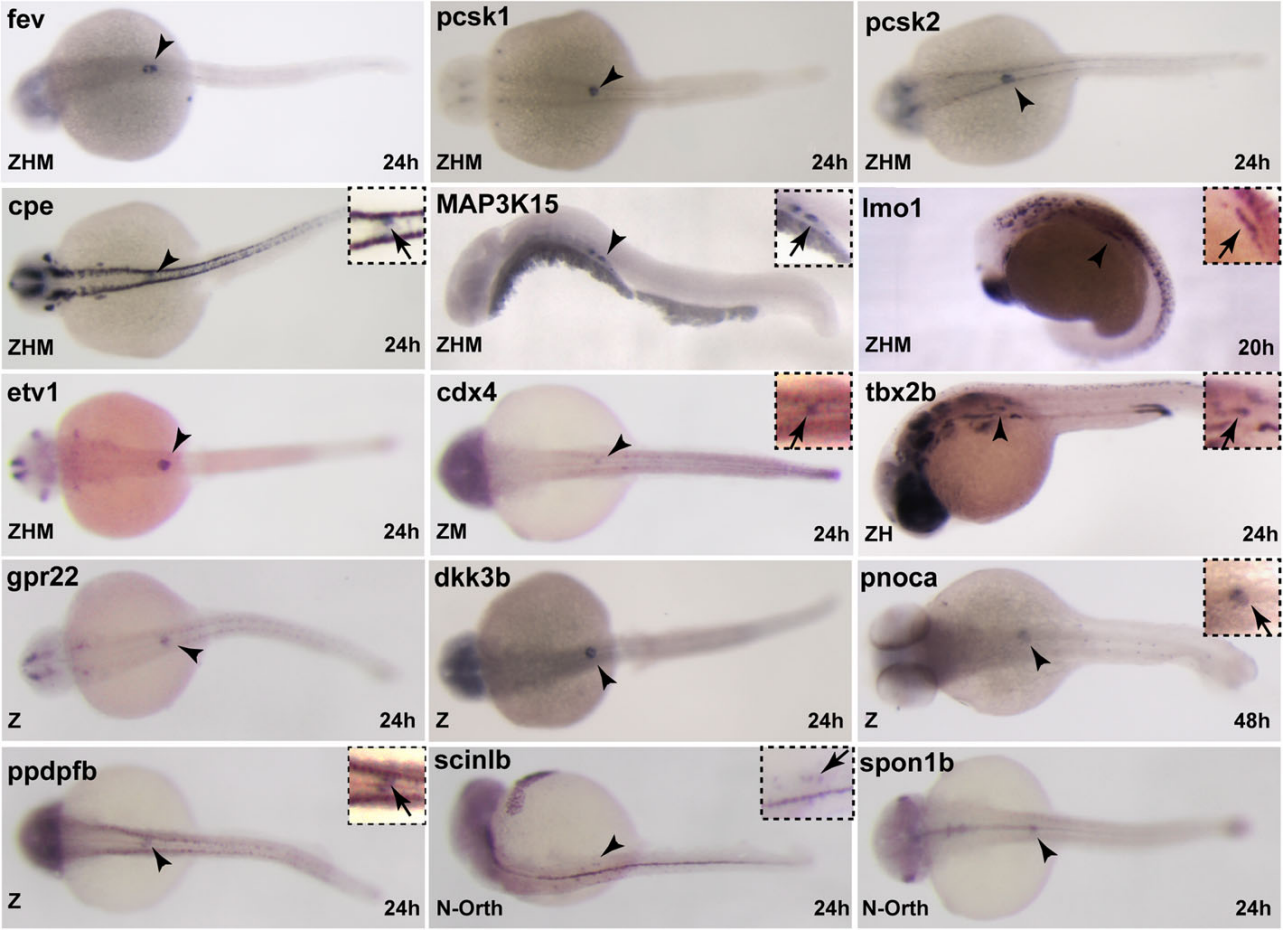
Expression of genes in endocrine cells of the dorsal pancreatic bud. Whole-mount in situ hybridization on zebrafish embryos showing endocrine pancreatic expression of some new zebrafish endocrine markers (n > 15). Genes with conserved endocrine-enriched expression in zebrafish, human, and mouse (ZHM), or endocrine-enriched in zebrafish and mouse (ZH), or in zebrafish and human (HM) are indicated. Z: Gene endocrine enriched only in zebrafish. N-orth: Endocrine enriched zebrafish gene with no obvious human or mouse ortholog. Arrowheads indicate the location of the dorsal pancreatic bud containing embryonic endocrine cells and insets at the top-right display higher magnification view of the pancreatic bud

While many transcription factors controlling pancreatic endocrine cell differentiation in embryos remain expressed and functional in mature cells from adults, there are nevertheless striking differences between the transcriptomes of fetal and adult cells [5]. In order to determine the fraction of the endocrine-enriched genes in adult zebrafish that are already expressed in the first embryonic endocrine cells, we determined the transcriptomic profile of endocrine cells isolated from *Tg(pax6b:GFP)* embryos at 27 hpf. We detected 9919 genes expressed in embryonic pancreatic cells above the threshold level of 100 Normalized count. By comparing these data with adult endocrine data, we found that, among the 1853 genes enriched in adult endocrine cells, 911 (49%) genes were already detected in embryonic cells (full gene list shown in Additional file 8: Table S6). The expression of some of these endocrine-enriched genes was further characterized by in situ hybridization (ISH) on whole zebrafish embryos (Fig. 4). We analyzed genes identified as endocrine enriched in the three species (the “ZMH” genes: *pcsk1*, *pcsk2*, *fev*, *cpe*, *etv1*, *map3k15*, and *lmo1*), in two species (the “ZM” gene *cdx4* and the “ZH” gene *tbx2b*), or only in zebrafish (the “Z” genes *gpr22*, *dkk3b*, *pnoca*, *ppdpfb*, *scinlb*, and *spon1b*). All these genes were detected in the embryonic endocrine pancreatic cells by ISH, validating the RNA-seq data. Thus, these data indicate that the early embryonic endocrine cells from the dorsal pancreatic bud express a large fraction of the genes constituting the adult endocrine signature.

### Characterization of the transcriptomic signatures for the endocrine cell subtypes

**Fig. 5:**
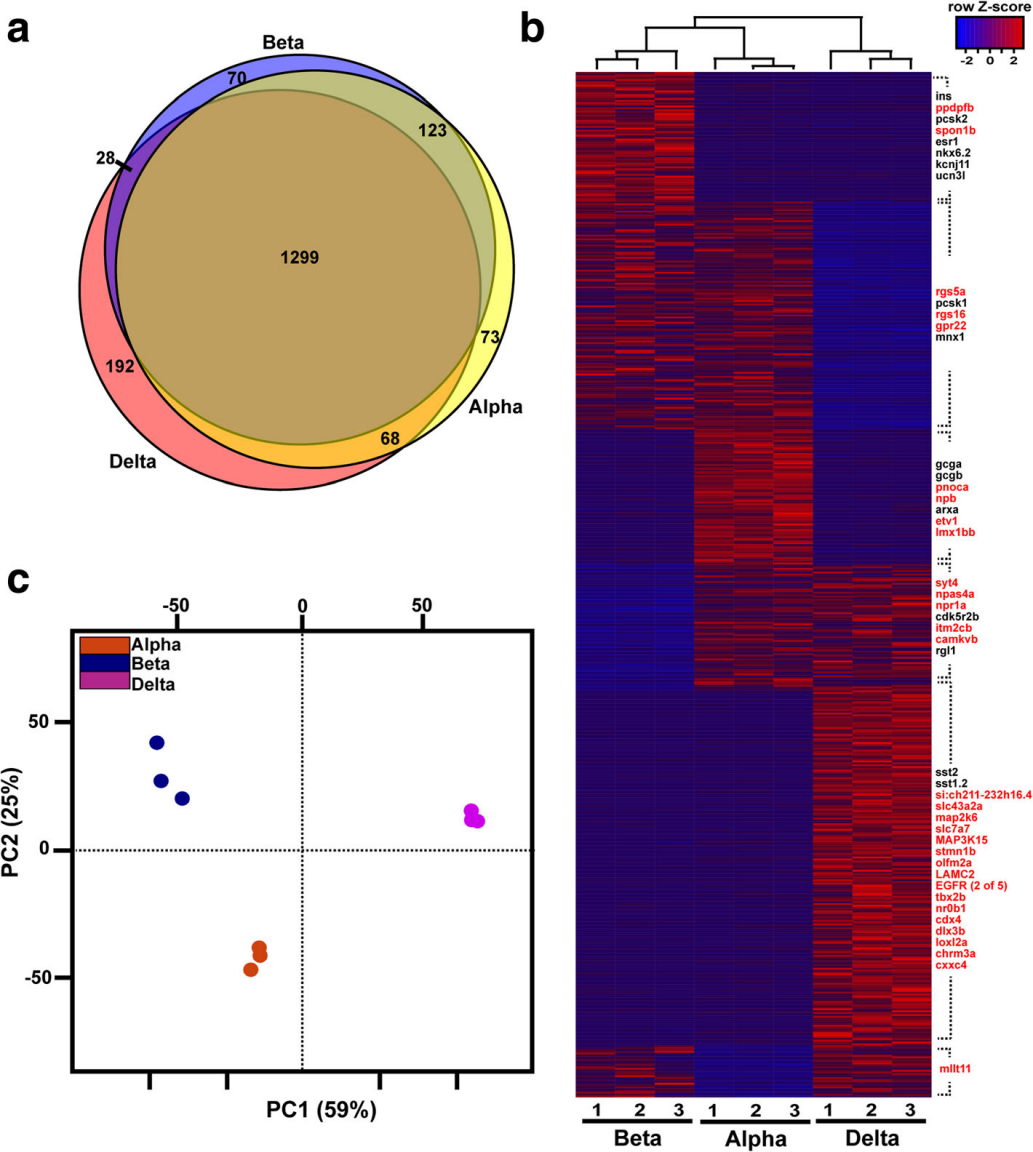
Zebrafish genes differentially expressed in the endocrine cell subtypes. **a** Venn diagram displaying the number of endocrine genes with alpha-, beta- and delta-enriched expression identified with DESeq algorithm based on a cut-off ratio of four-fold and adjusted *P* < 0.05. **b** Heatmap plot showing the expression pattern of all differentially expressed genes. Genes listed at the right side of the plot are some examples of either known (black) or new (red) markers identified in this analysis. **c** Principal component analysis performed on the nine zebrafish endocrine RNAseq datasets using the 1853 endocrine-enriched genes. Compared to the PC plot of Fig. 1b performed on all annotated zebrafish genes (33,726 genes), this plot shows a tighter clustering of all replicates and better discrimination between the three endocrine cell subtypes

**Fig. 6:**
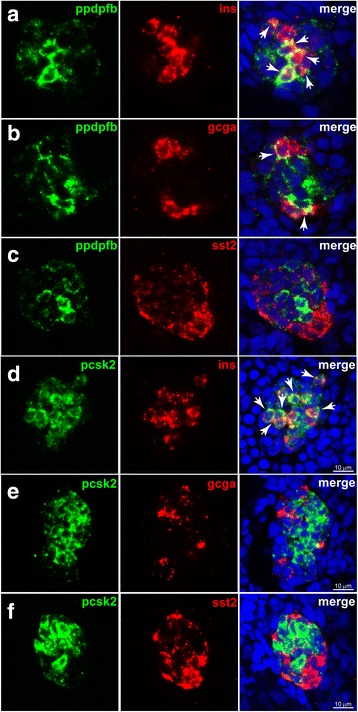
Expression of *ppdpfb* and *pcsk2* genes in zebrafish beta cells. Co-labeling by fluorescent *in situ* hybridization (FISH) of *ppdpfb* and *pcsk2* with insulin, glucagon, and somatostatin. **a**–**c** *ppdpfb* is mainly expressed by beta cells (**a**, arrows) and in few alpha cells (**b**, arrows). No expression of *ppdpfb* was observed in delta cells (**c**). Beta cells specifically expressed *pcsk2* (**d**, arrows) while no expression was detected in alpha or delta cells (**e** and **f**). (Analyzed embryos > 10)

In order to define the molecular signatures of alpha, beta, and delta cell subtypes, the 1854 endocrine genes identified in adult zebrafish (from Fig. 2a) were classified according to their differential expression in the distinct endocrine cell subtype (above the threshold of four-fold enrichment, adjusted *P* < 0.05); 73, 70 and 192 endocrine genes were found to be enriched in alpha, beta, and delta cells, respectively (Fig. 5a). The heatmap plot in Fig. 5b presents an overview of the expression pattern for all differentially expressed genes (gene list available in Additional file 9: Table S7). PCA using all 1854 endocrine-enriched genes displays a clear discrimination of the alpha, beta, and delta cell transcriptomes (Fig. 5c). GO enrichment analysis performed on beta cell-specific genes identifies “response to organic substrate”, “ion transport”, or “response to nutrient levels” as enriched pathways among others (Additional file 10: Figure S3A). The gene expressed at the highest level in the beta cells after *insulin* is *ppdpfb*. This gene is the paralog gene of the *ppdpfa* gene (*pancreatic progenitor cell differentiation and proliferation factor a*), which was reported as specifically expressed in acinar cells and controls their differentiation [44]. Interestingly, the paralog *ppdpfb* is specifically expressed in the endocrine pancreas and mainly in beta cells. We verified the expression of *ppdpfb* in 24 hpf zebrafish embryos by fluorescent ISH (FISH) and, according to the RNA-seq data, we confirmed its expression in many beta cells and in a few alpha cells but not in delta cells (Fig. 6a–c). The RNA-seq data also confirmed the selective expression of the zebrafish *nkx6.2* gene in beta cells, as previously reported [45]. Expression of the zebrafish *pcsk2* gene is more enriched in beta cells compared to the *pcsk1* gene (beta/alpha enrichment of 5.5- and 2.5-fold, respectively), while in mouse and human, only PCSK1 was reported to be more enriched in beta compared to alpha cells [4, 12, 46]. The enrichment of *pcsk2* in beta cells was confirmed by FISH on zebrafish embryos (Fig. 6d–f). The zebrafish estrogen receptor 1, *esr1*, is also strongly enriched in beta cells (10575 normalized counts in beta cells versus 671 in alpha and 71 in delta cells). Finally, the beta cell-selective expression of *spondin 1b* (*spon1b*), an activator of the Wnt pathway, was also validated by FISH on zebrafish embryos (Additional file 11: Figure S4).

As expected, the genes specifically expressed in alpha cells include those coding for the two zebrafish glucagon hormones (*gcga* and *gcgb*) as well as for the Arx transcription factor (*arxa*) but also novel markers including peptide hormones such as *prepronociceptin* (*pnoca*) and *neuropeptide B* (*npb*) or the calcium regulated factor scinderin like b (*scinlb*). The alpha-enriched genes also comprise the transcription factors *etv1* and *si:ch211-145o7.3* (a forkhead domain factor). The alpha-selective expression of *pnoca* and *scinlb* was confirmed by FISH, validating the RNA-seq data (Additional file 12: Figure S5).

As for the delta cells, besides the somatostatin genes [47], the RNA-seq data revealed many novel markers, which include genes coding for transcription factors such as *dlx3b*, *cdx1*, *cdx4*, and *tbx2b*, as well as genes coding for signaling factors like *bmp7b* or the kinase Map3k15. Interestingly, as demonstrated by the GO enrichment analysis (Additional file 10: Figure S3), many GPCR are specifically enriched in delta cells such as npy8br (neuropeptide Y receptor), glra4a (glycine receptor), uts2r (urotensin receptor), ptger1b & 2a (prostaglandin receptor), adra1d (adrenoreceptor), grm3 (glutamate receptor), oprd1b (opioid receptor), and gpr123, pinpointing these endocrine cells as targets of diverse external metabolic signals. We confirmed the selective expression for several genes, including the *cdx4*, *cdx1*, *lamc2*, and *map3k15* genes, detected already at 24 hpf in delta cells (Additional file 13: Figure S6). All these results validate the RNA-seq data and show that many endocrine cell subtype markers acquire their selective expression in the first embryonic endocrine cells.

### Comparison of transcriptomic signatures of alpha and beta cells across species

**Fig. 7:**
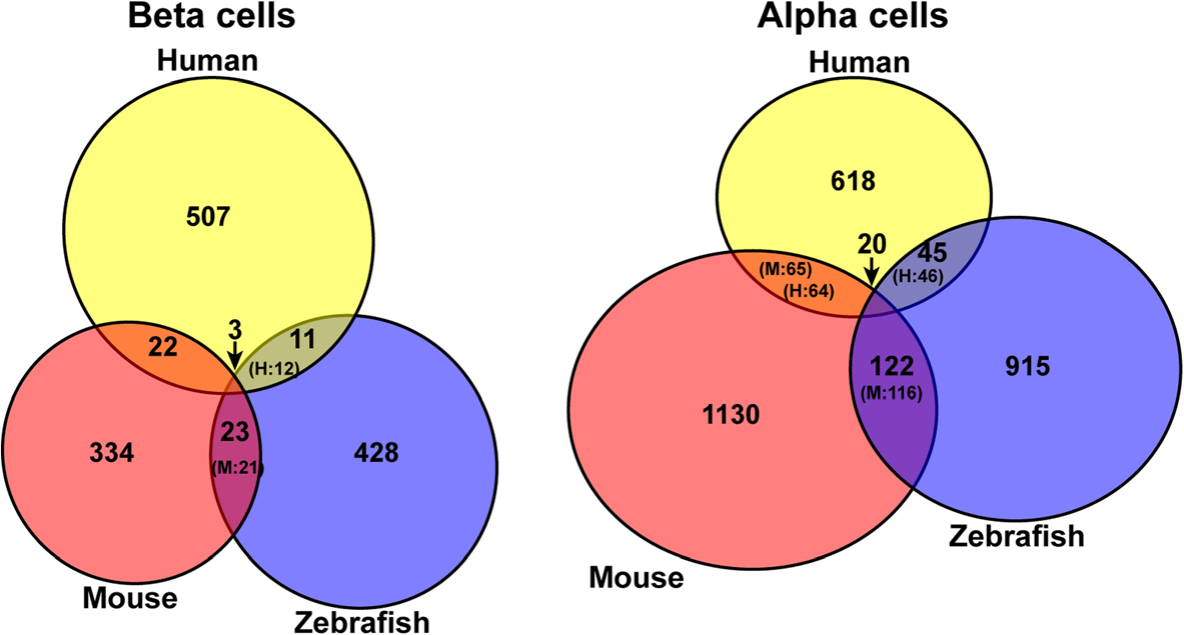
Identification of genes with conserved enriched expression in alpha and beta cells. Venn diagrams showing the number of genes presenting enriched expression in alpha cells (right panel) and beta cells (left panel) and displaying this enrichment in zebrafish, mice, and/or human (shown in intersections). Due to gene duplications in some species and often in zebrafish, the number of corresponding murine (M) or human (H) orthologous genes is given in brackets in each intersection. The alpha- and beta-enriched genes were selected by DESeq2 with fold change > 4 and adjusted *P* < 0.05 using two murine alpha and beta cell preparations [12], six human alpha and beta cell preparations [48], and three zebrafish alpha and beta cell preparations (this study). The full list of conserved beta- and alpha-enriched genes is given in Additional file 14: Table S8 and Additional file 15: Tables S9

The zebrafish RNA-seq data led to the identification of many novel markers for each endocrine cell subtypes raising the question of whether such markers display the same cell subtype-specific expression in other species. As the transcriptome of human and murine alpha and beta cells have been determined by a similar approach using highly purified FACS cell preparations [12, 48], this allowed us to perform an interspecies comparison. In order to identify all alpha and beta cell differentially expressed genes (with no restriction on endocrine enriched genes), we performed exactly the same procedure on the zebrafish, mouse, and human RNA-seq datasets, namely a comparison of the entire alpha and beta cell transcriptomes within each species using the same software and selection criteria (ratio of alpha vs. beta cells above four-fold enrichment, with adjusted *P* < 0.05). This led to the identification of 747, 1330, and 1102 alpha-enriched genes as well as 544, 381, and 465 beta-enriched genes in human, mouse, and zebrafish, respectively. Surprisingly, the interspecies comparison of these sets of genes revealed that the large majority of alpha- and beta- enriched genes are species specific (Fig. 7). Indeed, among the 465 zebrafish beta-enriched genes, only three have maintained beta cell preferential expression in human and mice, namely *insulin*, the transcription factor *pdx1*, and the glucagon receptor *gcgra* (Table 3 and Additional file 14: Table S8 for all ZH, ZM, and HM beta-enriched genes). Similarly, among the 1102 zebrafish alpha cell-enriched genes, only 20 have human and murine orthologs with alpha cell-enriched expression (Fig. 7, Table 3 and Additional file 15: Table S9). As expected, *glucagon* and *arx* are part of the conserved alpha cell genes. The *adcy2* and *adcy7* genes coding for adenylate cyclase are also alpha-enriched in the three vertebrate species supporting the important role of cAMP in alpha cells [49]. The *gc* gene (coding for vitamin D binding protein) [14] and *fev* (coding for a Ets transcription factor) are among the conserved alpha cell signatures, suggesting a specific function in this endocrine cell subtype. Taken together, all these analyses indicated that, while hundreds of genes display conserved expression in endocrine and exocrine cells, there is much less conservation for the genes differentially expressed between the endocrine cell subtypes alpha and beta. These data confirm the striking differences between species as previously reported for the human and murine alpha- and beta-enriched genes [12, 21]. Importantly, many of the conserved alpha- and beta-specific genes identified in the present study were also recently highlighted as enriched in these endocrine cell types in several single cell RNA-seq studies [19–21] (Additional file 14: Table S8 (beta cells) and Additional file 15: Table S9 (alpha cells) for comparison).

**Table 3:** List of genes with evolutionary conserved alpha and beta cell-enriched expression

Conserved beta cell-specific genes					
Zebrafish	Log2FC	Mouse	Log2FC	Human	Log2FC
pdx1	5.4	Pdx1	2.9	PDX1	6.4
gcgra	5.1	Gcgr	2.6	GCGR	4.2
ins	5.7	Ins2	5.4	INS	6.9
ins	5.7	Ins1	5.3	INS	6.9
Conserved alpha cell-enriched genes					
Zebrafish	Log2FC	Mouse	Log2FC	Human	Log2FC
arxa	12.1	Arx	6.7	ARX	6.0
nrxn3a	4.3	Nrxn3	3.1	NRXN3	2.5
adrb1	2.3	Adrb1	4.3	ADRB1	4.8
fap	2.4	Fap	3.0	FAP	7.1
adcy2a	6.0	Adcy2	5.7	ADCY2	3.2
grap2a	5.6	Grap2	2.3	GRAP2	2.4
ptprz1b	2.3	Ptprz1	5.0	PTPRZ1	4.6
gcgb	7.4	Gcg	5.6	GCG	7.5
gcga	5.5	Gcg	5.6	GCG	7.5
adcy7	3.5	Adcy7	3.7	ADCY7	2.2
adamts18	3.5	Adamts18	5.4	ADAMTS18	5.0
gata6	6.8	Gata6	3.0	GATA6	4.3
gc	4.9	Gc	2.5	GC	6.6
fev	4.3	Fev	5.0	FEV	5.5
tgfbr2	5.6	Tgfbr2	2.6	TGFBR2	2.3
kcnc2	7.8	Kcnc2	4.0	KCNC2	4.9
fgb	2.9	Fgb	2.5	FGB	3.0
marcksl1a	5.4	Marcksl1	2.7	MARCKSL1	2.1
mrc1b	5.5	Mrc1	2.2	MRC1L1	2.9
tgm2b	4.2	Tgm2	2.6	TGM2	2.1

Expression enrichment in alpha and beta cells is given as the Log2 fold-change in the three species

### Role of the zebrafish *myt1b* and *cdx4* genes in endocrine cell differentiation

**Fig. 8:**
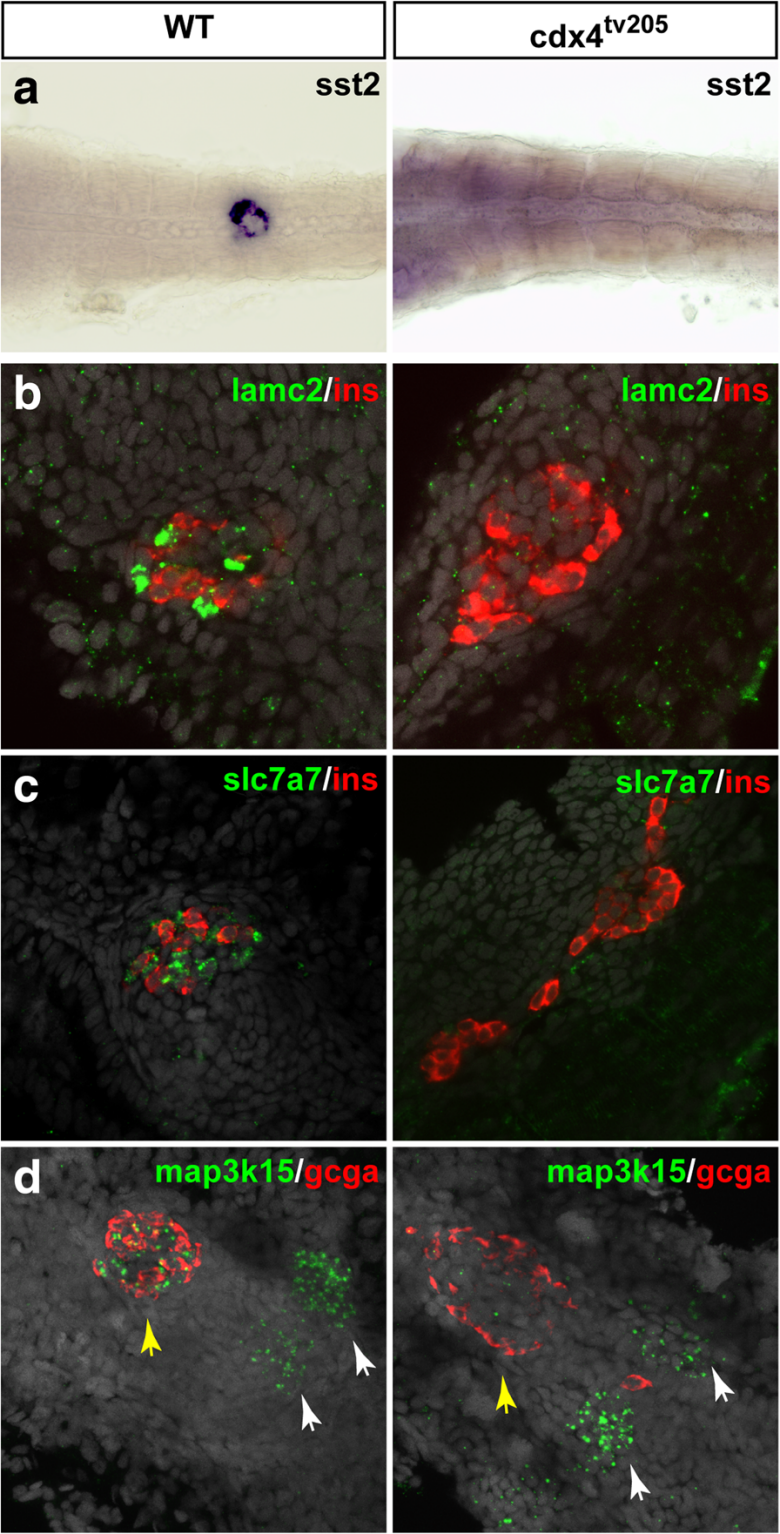
Delta cell differentiation is disrupted in the cdx4 mutant embryos. Analysis of wild-type and *cdx4* ^tv205–/–^ mutant embryos at 48 hpf by WHISH (**a**) and FISH (**b**–**d**) using delta cell markers (*sst2*, *lamc2*, *slc7a7*, and *map3k15*) as well as *gcga* and *insulin. cdx4* ^tv205^ mutants display a loss of *sst2* (**a**), *lamc2* (**b**), and *slc7a7* (**c**) pancreatic expression and an increase of beta cells*. Map3k15* expression is strongly reduced in the pancreatic islet (yellow arrows) while not affected in the presumptive pronephric glomeruli (white arrows) (**d**). No obvious effect is observed on *gcga* expression. Nuclei are stained with DAPI (grey staining). (Analyzed embryos > 10)

To validate our cross-species approach to identify important pancreatic regulatory factors, we selected two transcriptional regulators that were expressed at high levels in endocrine cells at the early developmental stage (27 hpf) and performed loss of function studies. Mutations of *myt1b* gene (“ZMH” conserved endocrine genes) and of the *cdx4* gene (“ZM” conserved endocrine gene) were found to affect endocrine cell differentiation. As *cdx4* is selectively expressed in delta cells, we analyzed the expression of several novel delta cell markers in the *cdx4* ^tv205^ null zebrafish mutant [50]. A previous study has shown that the zebrafish *cdx4* mutant displays defects in the anterio-posterior patterning of the endoderm, with notably a posteriorly-shifted pancreas and an increase of pancreatic beta cell number [51]; however, the selective expression of *cdx4* in delta cells was unknown as well as its function in this endocrine cell type. Analysis of the null *cdx4* ^tv205^ mutant embryos revealed a complete loss of *somatostatin2* gene expression (Fig. 8a). Similarly, expression of the new delta cell markers *lamc2* and *slc7a7* was almost undetectable in the mutant embryos, in contrast to the increase of insulin expression (Fig. 8b, c). Expression of *map3k15* gene in delta pancreatic cells was also specifically abrogated by *cdx4* mutation, while expression of this gene was not affected anteriorly at the level of the pronephric glomeruli (white arrows Fig. 8d). All these data demonstrate the important role of *cdx4* for delta cell differentiation.

**Fig. 9:**
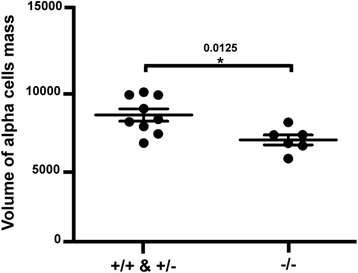
Reduced glucagon expression in the *myt1b* zebrafish mutant embryos. Glucagon expression was analyzed by immunofluorescence in F2 *myt1b* ^*ulg019*^ homozygous mutant larvae and in sibling ^+/+^ (WT) and ^+/–^ larvae at 2 dpf. The volume of the alpha cell mass was determined in each embryo by the imaging software Imaris. The graph shows the quantification measured for all myt1b^–/–^ embryos and for ^+/–^ or ^+/+^ siblings, indicating a slight by statistical significant decrease of alpha cell mass (each point is the alpha cell mass measured in one embryo)

Our transcriptomic analyses show that embryonic pancreatic cells express both *myt1a* and *myt1b* paralogs, with *myt1b* being expressed at much higher levels*.* This was confirmed by ISH revealing expression of *myt1b* in the dorsal pancreatic bud of zebrafish embryos (Additional file 16: Figure S7), while the paralog *myt1a* was barely detectable (data not shown). The adult RNA-seq data indicate that the *myt1a/b* genes are expressed in alpha, beta, and delta cells. Simultaneous inactivation of the two zebrafish *myt1* genes was performed by multiplex CRISPR/Cas9 mutagenesis through injection of four guide RNA (two different guides targeting each gene, see Methods). Injected embryos revealed a significant decrease of *glucagon* expression and no significant effect on *insulin* expression (Additional file 16: Figure S7). To confirm these data obtained in F0 embryos, a line harboring a null mutation in *myt1b* (*myt1b* ^*ulg029*^) was raised. Alpha cell mass was decreased in the *myt1b* ^*ulg029*–/–^ embryos compared to the wild-type and heterozygous siblings (Fig. 9).

## Discussion

In this study, we have explored the transcriptomic landscape of the major pancreatic cell types in zebrafish, thereby defining the transcriptomic signature of acinar and endocrine cells, as well as of the three major endocrine cell subtypes alpha, beta, and delta. By this analysis, we identified many novel cell type-specific markers with still unknown pancreatic function. By comparing the endocrine and exocrine transcriptomic signatures from zebrafish, human, and mouse, we could define an evolutionarily conserved signature for these two pancreatic tissues, pinpointing genes and pathways that likely represent key players in the pancreas of vertebrates. Consistent with this notion, more than half of all transcription factors in these endocrine or exocrine conserved signatures are known regulators of pancreatic cell differentiation, such as the transcription factors *neuroD*, *isl1*, *pax6*, *insm1*, *ptf1a*, and *rbpjl*, among others. *Myt1* is part of the endocrine conserved signature and we show here that inactivation of the *myt1b* gene in zebrafish leads to a decrease of alpha cell mass in 2 dpf embryos. Additionally, *cdx4*, which is selectively expressed in delta cells, is also necessary for their differentiation.

The endocrine signature conserved among the three vertebrate species reveals that several signaling pathways regulating hormone secretion and cellular homeostasis are commonly used by pancreatic cells from fish to mammals. For example, the evolutionarily conserved signature comprises the RNA binding protein *Elavl4/Hud* and the ionotropic glutamate receptor *Gria2*, shown to regulate hormone synthesis and secretion in rodents [40, 52, 53]. Similarly, the glucose response system regulating insulin and glucagon secretion in mammals seems to be also used in zebrafish as various components of this pathway are found in the conserved endocrine genes such as the ATP sensitive K^+^ channels *abcc8* and *kcnj11* and the voltage-dependent Ca^++^ channel *cacna2d2*. The use of this pathway in zebrafish is further strengthened by the very high expression of the glucose transporter glut2 (*slc2a2*) and the glucokinase (*gck*) in zebrafish beta-endocrine cells. Additionally, some GPCR receptors which have been reported to control the activity of pancreatic islet cells in mice, such as the Sstr3 (somatostatin receptor) [54], CasR [55], or Celsr3 [56], are included in the conserved endocrine signature. Thus, this list of conserved endocrine-enriched genes strongly suggests that the function of several signaling pathways controlling the formation and activity of pancreatic cells has been maintained from fish to humans. This is consistent with many studies using zebrafish as a model for pancreas development and diabetes that revealed conserved physiological regulations (reviewed in [34, 35]). The conserved endocrine and exocrine pancreatic signatures also pinpoint to many novel candidate regulatory genes that deserve special attention for future studies. For instance, the transcription factors *lmo1* and *npas4a*, or the signaling factors *gpr158*, *rgs7*, and *rgs17*, all selectively expressed in pancreatic endocrine cells in zebrafish, mice, and human, more than likely play an important role in pancreatic cells.

In the present study, we identified the human and murine endocrine- and exocrine-enriched genes by comparing transcriptomic data from purified islets with data obtained from whole pancreas as well as from one human pancreatic sample enriched in acinar tissue. While this approach identified almost all expected endocrine and exocrine markers, some markers may have been missed due to the low purity of tissue preparations. For example, the human *MNX1* and murine *Esr1* (*Estrogen receptor alpha*) genes were not enriched in the human or murine endocrine samples, respectively, while these two genes were reported to be important for endocrine beta cells in both species [57–60]. These misclassifications may be due to the use of RNA-seq data obtained from whole pancreas instead of highly purified exocrine cells; such potential errors will be corrected when RNAseq data from purified ductal and acinar cells will be available. The recent single cell transcriptomic data reported for human and murine pancreas bypass the need for purified cells and will probably constitute a useful resource for doing such interspecies comparison. However, murine acinar cells have not been captured in these single-cell studies preventing the identification of acinar-enriched and endocrine-enriched genes. When such data is available, it will be interesting to perform a global analysis of all pancreatic single cell transcriptomic studies and compare it with the zebrafish pancreatic data. In the meantime, genes classified in our list of conserved “ZM” or “ZH” endocrine genes should be also considered as they indeed include *mnx1* and *esr1*, indicating that important genes fall in these two categories. For proof, *cdx4*, classified as endocrine-enriched in zebrafish and mice (i.e., “ZM”), was found here to be crucial for endocrine delta cell differentiation as many novel delta cell makers were drastically reduced in the *cdx4* mutant zebrafish embryos. Our findings warrant future analyses on the murine (and human) *Cdx4* gene to decipher its expression and function during pancreas development in mammals. Interestingly, the loss of delta cells with the concomitant increase of beta cells observed in the zebrafish *cdx4* mutant suggests that Cdx4 could act on the balance of delta versus beta cells by determining the fate of endocrine precursors.

The present study also provides a comprehensive list of new markers of the zebrafish endocrine alpha, beta, and delta cell subtypes. An overall view indicates that the alpha and beta cells are slightly more similar to each other compared to delta cells, as shown by the PC analysis (Figs. 1b and 5c). This is also supported by the Venn diagram (Fig. 5a) showing that (1) the number of genes with enriched expression in both alpha and beta cells and not in delta cells (“alpha-beta” enriched genes) is higher than those of the “alpha-delta” enriched and “beta-delta” enriched genes (123 genes vs. 28 and 68 genes, respectively), and (2) the number of delta cell markers is higher than the alpha and beta cell marker (192 vs. 70 and 73 genes, respectively). These observations are consistent with a recent study in zebrafish larva showing the higher capacity of alpha cells to transdifferentiate to beta cells compared to delta cells [61]. Whether this is also true in adult zebrafish must be verified as it has been demonstrated in mice that the transdifferentiation competence of alpha or delta cells toward beta cells is age dependent [62].

An unexpected observation of our study is the low conservation of the alpha and beta cell transcriptomic signatures between zebrafish, mice, and human. Indeed, only 20 and three genes defined the conserved alpha and beta cell signatures, respectively. Such low conservation cannot be attributed to a low quality of cell samples or RNA-seq data because, for the three species datasets, there is a very high enrichment of alpha and beta cell markers in the corresponding libraries confirming the good purity of alpha and beta cell preparations. Further, it is noteworthy that this low conservation is not only observed between zebrafish and the two mammalian species but also between human and mouse (see Venn diagram in Fig. 8). Differences between human and mouse alpha- and beta-enriched genes have also been previously noticed [12, 21]. Several non-exclusive explanations can be proposed for this apparent low conservation of the alpha- and beta-signatures in both distant and closer species. First, as the alpha and beta cells have relatively similar transcriptomes, as shown on Fig. 1a, the identity of these two cell subtypes could rely on the action of only few regulatory genes. This hypothesis is supported by the presence of *Arx* among the few conserved alpha cell genes, which is the key determinant of alpha cell identity and its inactivation in mice is sufficient to transdifferentiate alpha to beta cells [63]. Similarly, Pdx1, one of the three conserved beta cell genes, is required to maintain beta cell identify and repress the alpha cell program [64]. This first hypothesis is also supported by the ability of alpha and beta cells to transdifferentiate into each other [65, 66]. A second explanation can be the relatively stringent threshold that we used to select differentially expressed genes (four-fold enrichment, adjusted *P* < 0.05). For example, the *mnx1* gene, known to be involved in beta cell differentiation [67, 68], is enriched in beta cells in the three vertebrate species but was not classified as beta-enriched in human and zebrafish due to a rather low beta versus alpha cell enrichment (three-fold in human, 1.9-fold in zebrafish versus 70-fold in mice). These differences in enrichment between human and mouse *Mnx1* is confirmed in the recent single cell RNA-seq data [18–21]. Similarly, the *prohormone convertase 1* gene *pcsk1* required for insulin hormone maturation was only 2.7-fold enriched in zebrafish beta cells versus alpha cells. It is possible that small differences in expression levels may be sufficient for some genes to give a different physiological response. A third explanation of the low cell subtype conservation could stem from functional switches occurring between homologous genes able to perform the same function. For example, *Nkx6.1* is expressed in beta cells in mice and human but not in zebrafish, where its paralog *nkx6.2* instead of *nkx6.1* is selectively expressed in mature beta cells [45]. *nkx6.2* may fulfill the function of *Nkx6.1* as Nkx6.1 and Nkx6.2 have equivalent biological activities [69]. Another example is the Zn^++^ transporter *scl30a8*, which is strongly expressed in beta cells in mammals but not in zebrafish, which instead strongly expresses the homologous Zn^++^ transporter *scl30a2*. Such functional switches may be difficult to identify as they can occur between genes of the same superfamily but belonging to different subclasses. For example, we have shown that, in zebrafish, the role of the bHLH Neurog3 as pancreatic endocrine cell fate determinant is fulfilled by two other bHLH factors Ascl1b and Neurod1 [70]. A functional switch is also observed for the specification of the secretory cell of the intestine, played by Atoh1 in mice and by Ascl1a in zebrafish [71]. The capacity of homologous factors to fulfill the same function is also supported by the observations that many null mutations can be compensated by a homologous gene [72–74]. Such compensations seem to occur for the murine *Myt1* gene as the *Myt1* KO mice only display very mild pancreatic defects with no decrease of endocrine cells [75], while more drastic defects are observed by expression of a dominant negative Myt1-Eng protein [1, 76]. Furthermore, adult murine endocrine pancreatic cells express the homologous *Myt1l* and *Myt3/St18* genes [77] and their expression increases in the *Myt1* KO mice [75]. In zebrafish, *myt1a*, *myt1l*, and *myt3/st18* are expressed at much lower level compared to *myt1b*; this probably explains the decrease in alpha cell mass observed in the single *myt1b* mutant. Further experiments will be required to determine whether some compensation occurs through *myt1a* and if the defects are more drastic in the double *myt1a/b* mutant.

The stronger expression of glucagon receptor in the beta cells of the three vertebrate species highlights the importance of paracrine regulations between alpha and beta cells and indicates a role of glucagon on beta cell physiology, as previously reported in rodents [78] and in zebrafish for beta cell regeneration [61]. Interestingly, our transcriptomic analyses highlight *fev* as an endocrine conserved gene as well as an alpha cell conserved gene, suggesting a role of this transcription factor in alpha cells. Glucose tolerance test in *Fev* knockout mice previously revealed a slower response apparently due to a reduction in insulin production while the level of most crucial transcription factors of beta cells were unchanged [79]. As our transcriptomic analysis indicates that *fev* is expressed more than 20-fold higher in alpha cells versus beta cells in zebrafish, mice, and human, this argues for an important function in alpha cells and warrants further phenotypic analyses.

## Conclusions

The present transcriptomic analysis of the distinct zebrafish pancreatic cell types identifies novel pancreatic regulatory genes and thereby constitutes a valuable resource for future studies of the pancreas not only in zebrafish but also in mammals giving interesting clues on genes and signaling pathways active in these cells. The comparison of the present zebrafish pancreatic RNA-seq data with the recent single cell pancreatic transcriptomic data or with future data of pure pancreatic cells obtained not only from human and mouse but also from other species will help to better define gene regulatory networks controlling pancreas ontogeny and physiology in vertebrates. This will be useful for studies aimed at understanding dysfunction of pancreatic endocrine cells in human diseases like diabetes and to design novel drugs or therapies.

## Methods

### Generation of the Tg*(Insulin:GFP)*^*ulg021 Tg*^*line*

The *(Insulin:GFP)* transgene was generated by first cloning a 897 pb PCR fragment, amplified with O97 and O98 primers (Additional file 17: Table S10), that includes 745 bp of the insulin promoter, the exon 1 and intron 1 and 7 bp of exon 2 just upstream of the ATG of the insulin ORF, into the gateway vector pCR8/GW/TOPO to produce pMV90-G2a plasmid. Second, a triple LR recombination using p5E-MCS, pMV90-G2a, and p3E-EGFP-PA inserted into pDestTol2pA2, provided by the tol2kit [80], generated the transgene *Tg(insulin:GFP)* that has been introduced into AB embryos by coinjection with the Tol2 transposase to generate the *Tg(insulin:GFP)* ^*ulg021 Tg*^ line.

### Preparation of zebrafish pancreatic cells by FACS

The endocrine cells were prepared using the zebrafish transgenic lines *Tg(Insulin:GFP)* ^*ulg021Tg*^, *Tg(gcga:GFP/ins:mCherry)ia1*, and *Tg(st22:GFP)* to isolate beta, alpha, and delta cells, respectively [28, 81]. Acinar and ductal cells were isolated by using *Tg(ptf1a:GFP)* [27] and *Tg(nkx6.1:GFP)* ^*ulg004Tg*^ [29], respectively. The endocrine tissue was dissected under an epifluorescence stereomicroscope before dissociation by enzymatic treatment for 30 min at 28 °C with 1× Tryple Select (Life Techologies), 40 μg/mL proteinase K (Roche), and 10 μg/mL collagenase IV (Life Technologies), combined with mechanical disruption by pipetting every 5 minutes. For acinar cell preparations, pancreata were digested with a mix of collagenase IV (500 μg/mL), collagenase P (350 μg/mL), and dispase II (1 mg/mL) for 15 min at 28° with mechanical disruption every 5 minutes. After dissociation, cells were washed twice with 1× PBS containing 1% BSA and pelleted at 4 °C for 5 min at 300 *g* and immediately sorted by FACS. Cells were selected based on GFP expression using FACS Aria II by two consecutive sorting steps: the first sorting was done in the “yield” mode and the second in the “purity” mode. Purity was estimated by FACS Aria II after cell sorting (more than 99% purity of GPF^+^ cells) and by fluorescence microscopy (from 95–99% purity). Each replicate sample was prepared from four adult zebrafish. Approximately 10,000–20,000 endocrine cells, 20,000–40,000 acinar cells, and 2000–10,000 ductal cells were obtained after FACS and used for library preparations.

### RNA extraction, cDNA amplification, library preparation, and sequencing

Total RNA was extracted from FACS sorted cells using the RNeasy plus micro kit (Qiagene). RNA from endocrine and acinar cells was eluted in 10 μL with a concentration of 100–400 pg/μL. RNA integrity was assessed by a capillary electrophoresis using Agilent RNA 6000 pico chip (Agilent technologies), the RIN value for each sample was from 8 to 10. The Smarter Ultra low RNA input kit (clontech) [82] was used to for the synthesis and amplification of cDNA synthesis using up to 10 ng of total RNA following the manufacturer’s instructions and performing no more than 12 cycles of PCR in order to minimize amplification biases. The quality of cDNA was verified by 2100 High Sensitivity DNA assay (Agilent technologies). Truseq DNA Illumina libraries were prepared and sequenced to obtain approximately 90 million reads (100 bp paired-end reads) per library using the Hiseq 2000 Illumina sequencer.

### RNA-seq data analysis

Sequences were trimmed in order to remove adaptors and low quality bases. Trimmed reads were mapped in to the genome (Zv9, Ensembl genome version 75) using Tophat v.2.0.9 [83]. Tophat’s options were set according to the library features (-r 220 --mate-std-dev 82 --segment-length 18) and the option --min-intron-length was set up to 30 nucleotides according to the intron length described by Moss et al. [84]. For the mouse and human datasets, raw data were downloaded from the public databases: human pancreas (four samples from Fagerberg et al*.* [37] and one acinar cell-enriched sample from Morán et al. [9]: the E-MTAB-1294 dataset), human islets (three samples from Nica et al*.* [46] and four samples from Morán et al*.* [9]), murine pancreas (two samples from Holmstrom et al*.* [36]), and murine islets (three samples from Morán et al*.* [9]). The alpha and beta cell RNA-seq datasets were obtained from Benner et al. [12] for mouse (two samples of each) and from Blodgett et al. [48] for human (six samples each) [48]. Reads were mapped into the mouse GRCm38 and the human GRCh37 genomes (Ensembl genome version 75) using default options. Gene expression was measured from the mapped reads by using HT-seq-count [85]. PCA, using princomp function of R, was calculated for the whole dataset using the variance stabilization transformation values obtained by DESeq R package from the gene expression values [86]. For differential expression analysis, we used the R package DESeq2 [87]. DESeq2 employs shrinkage estimation for dispersions and fold change; it uses Wald test for significance with posterior adjustment of *P* values using the procedure of Benjamini and Hochberg (giving adjusted *P* values). Genes differentially expressed were selected with an adjusted *P* < 0.05 and a fold change > 4. For the comparison of the acinar, ductal and endocrine cell data, in-silico endocrine datasets were simulated by combining alpha, beta and delta RNA-seq data. This approach was taken to decrease the number of pairwise comparisons and could be used since the sequencing deepness of each library was in a similar range (below of a factor 2). Endocrine dataset #1 was obtained by combining the mapped reads of alpha1 (47 × 10^6^ reads), beta1 (48 × 10^6^ reads), and delta1 (31 × 10^6^ reads) datasets; endocrine dataset #2 is a mix of alpha2 (46 × 10^6^ reads), beta2 (29 × 10^6^ reads), and delta2 (30 10^6^ reads); endocrine dataset #3 is the combination of alpha3 (59 × 10^6^ reads), beta3 (62 × 10^6^ reads), and delta3 (59 × 10^6^ reads) datasets. The RNA-seq raw data have been deposited on ENA under the accession number PRJEB10140.

### Comparison of the human, murine, and zebrafish pancreatic transcriptomes

The predicted orthologs among zebrafish, mouse and human were obtained from Ensembl [88]. The information from the three species was retrieved using Biomart tool [89]. Two different orthology tables were generated (tables available upon request). The first table contains all zebrafish, murine and human genes with 1-1-1 orthology relationship. Orthology table two comprises all 1-1-1 orthologs as well as the genes presenting 1-many-many orthology relationships and thus includes all duplicated genes (paralogs), which are notably found in the zebrafish genome. The interspecies PC analysis was performed using only the genes with a 1-1-1 orthology relationship. The genes presenting an endocrine-enriched or an exocrine-enriched expression were identified in mouse and in human by using DESeq2 software selecting genes with at least four-fold higher expression in islet dataset or in whole pancreas dataset (adjusted *P* < 0.05). The interspecies comparison of endocrine- and exocrine-enriched genes was performed using the orthology table two.

### GO enrichment analysis

Tissue- and cell type-enriched genes were converted and uploaded to DAVID bioinformatics resource [90]. Using the Functional annotation tool, we run a GO enrichment analysis for endocrine and acinar enriched genes as well as for the alpha-, beta- and delta-enriched genes. Enriched processes were identified by GOTerm_BP_FAT with a *P* < 0.1.

### In situ hybridization

Antisense RNA probe for the different genes were prepared as described by Thisse et al. [91], except for *fev* [92]. Briefly, primers were designed to amplify a part of the transcript that is used as a template to synthesize the probe. The reverse primer at the 5’ end contains the minimal promoter sequence for T3 RNA polymerase (5’-AATTAACCCTCACTAAAGGGAG-3’), templates were amplified by RT-PCR using the set of primers shown in Additional file 17: Table S10. Whole mount in situ hybridization and fluorescent in situ hybridization (WISH and FISH) were performed as described by Mavropoulos et al. [93], applying some modification to this protocol. Briefly, larvae of 3 dpf or older were incubated during 20 minutes in methanol and 3% H_2_O_2_ at room temperature, prior to dehydration. Antisense probe hybridization was performed using 10–50 ng of DIG- and DNP-probes in hybridization buffer containing 5% dextran sulfate (MW: 500,000) at 65 °C overnight. Antibodies were pre-absorbed on homogenized larvae (mix of different developmental stages) for 2 h at room temperature and then diluted to 1/3000 DIG-AP, 1/1500 DIG-HRP, and 1/800 DNP-HRP (PerkinElmer).

### Inactivation of *myt1a* and *myt1b* genes by multiplex CRISPR/cas9 mutagenesis

Mutations in the *myt1a* and *myt1b* genes were generated by multiplex CRISPR/Cas9 technology essentially as described previously [94, 95]. The nls-zCas9-nls mRNA was synthesized by transcription of the plasmid pT3TS-nCas9n (Addgene). CRISPR guide RNAs were selected using CRISPR design and chopchop software to target the beginning of Myt1a and Myt1b coding regions (guides 1) and the regions coding for the first zinc finger domain (guides 2). The following target sites were used: GCCAAGACGCAGATGATAAGCGG and GATGGTTTAGGCCATGTCAGTGG for *myt1a*, and GTCTGAGGGAGGGCCGGCAGCGG and TGCCATTGCATCCTGGAGTGGGG for *myt1b* (PAM motifs are underlined)*.* The DNA templates were prepared by annealing and filling two oligonucleotides containing the T7 promoter sequence and the target sequences as previously described [95]. After synthesis and purification of gRNA, fertilized zebrafish eggs were injected with approximately 1 nL of a solution containing 50 ng of the four gRNA and 300 ng of nls-zCas9-nls mRNA. The efficiency of mutagenesis was verified by genotyping using Heteroduplex Migration Assays after amplification of targeted genomic sequences. A few injected embryos were fixed in PFA at 48 hpf for phenotypic analysis and the others were raised until adulthood. Founder fish transmitting a germline mutation in myt1b were outcrossed with wild type fish; F1 fish harboring a 5 bp insertion in *myt1b* (*myt1b* ^*ulg039*^ allele) causing a frameshift in the coding sequences were incrossed to generate *myt1b* ^*–/–*^ embryos and ^+/–^, ^+/+^ siblings, which were analyzed by immunohistochemistry.

### Immunohistochemistry

Expression of insulin and glucagon was analyzed by immunofluorescence on whole-mount zebrafish embryos. After overnight fixation in 2% PFA at 4 °C, 1 hour incubation in PBS 1% Triton X-100 for 2 hours at room temperature in blocking solution (4% BSA, 10% DMSO, 0.3% Triton X-100 in PBS), embryos were incubated with the primary antibodies anti-Mouse Glucagon 1/200 (Sigma, G2654) and Anti-Guinea Pig Insulin 1/300 (MP, 64714). After washing, embryos were incubated with the secondary antibodies Anti-Mouse Alexa 568 or 633 (Invitrogen, A-11004 and A-21052) and Anti-Guinea Pig Alexa 568 or 633 (Invitrogen, A-11075 and A-21105) diluted 1/300. After washing and mounting, embryos were scanned with a Leica SP5 confocal microscope and images were analyzed using Imaris 7.2.3 software. Alpha and beta cell mass was measured using Imaris based on 3D reconstitution of Fluorescence signal obtained by immunofluorescence; the same confocal and Imaris parameter settings were used for wild-type and mutant embryos. To compare the number of alpha cells and beta cells in wild-type and mutants, glucagon^+^ and insulin^+^ cells were counted in every 6-μm optical section throughout the whole principal islet. Expression of GFP and mCherry in the pancreas of Tg(gcga:GFP);(ins:NTR-mCherry) adult transgenic fish as well as of Tg(sst2:GFP) adult fish was analyzed by immunofluorescence on cryosections (Additional file 1: Figure S1). The antibodies used are the same than described above and also include the anti-Rabbit Somatostatin 1/300 (Dako, a0564) and Anti-Rabbit Alexa 568 or 633 1/300 (Invitrogen, A-11011 and A-21070). Expression of GFP in beta cells was also verified for the Tg(insulin:GFP) larvae (Additional file 1: Figure S1).

## Additional files

Additional file 1: Figure S1. Expression of the transgenes *Tg(ins:GFP)*, *Tg(gcga:GFP)*, *Tg(sst2:GFP)*, and *Tg(ins:NTR:mCherry)* in the endocrine pancreatic cell types. (A–C) Whole mount immunostaining of 3 dpf transgenic larvae *Tg(ins:GFP)* demonstrating the selective expression of GFP in beta cells. (D–I) Immunostaining of pancreas sections from adult transgenic fish *Tg(gcga:GFP/ins:NTR:mCherry)*. Glucagon^+^ cells (see arrows in D–F) express high level of GFP and are not labelled by mCherry, while many insulin expressing cells are labelled by mCherry and by GFP (at slightly lower levels) (see arrowheads in G–I). These data reveal a leaking expression of the gcga:GFP transgene in beta cells. (J–L) ISH performed on pancreas section of adult Tg(sst2:gfp) with sst2 probe followed by immunofluorescence using the GFP antibody; the expression of endogenous sst2 gene co-localize with GFP staining confirming the specific expression of the transgene in delta cells [28]. (TIF 8638 kb)
Additional file 2: Table S1. Gene expression levels in alpha, beta, and delta cell subtypes. The expression is given in Normalized counts for the three replicates of alpha, beta, and delta cell libraries. (XLSX 4416 kb)
Additional file 3: Table S2. Gene expression levels in endocrine, acinar, and ductal cells. The expression is given in Normalized counts for the four replicates of acinar cells, the three replicates of ductal and endocrine cells. The three endocrine datasets were obtained by combining the reads obtained with alpha, beta and delta cell libraries as described in Methods (e.g., endocrine1 data is a mix of alpha1, beta1 and delta1; endocrine2 data is a mix of alpha2, beta2 and delta2) (XLSX 4433 kb)
Additional file 4: Table S3. Classification of genes according to their enriched expression in endocrine, acinar, or ductal cells. Expression levels were compared for each gene between endocrine, acinar, and ductal cells (pair-wise comparison with DEseq2). Classification was performed using the cut-off values of fold change > 4 and adjusted *P* < 0.05. The enrichment is given for each gene as Log2 of fold change and with the adjusted *P* value. (XLSX 2192 kb)
Additional file 5: Table S4. Genes presenting conserved endocrine expression. The ZMH excel page shows the genes with endocrine-enriched expression in zebrafish, mice and human. The ZM, ZH and HM excel pages show genes with endocrine-enriched expression in two species (ZM: zebrafish and mice; ZH: zebrafish and human; HM: human and mice). The tables give the expression mean in endocrine cells (Normalized counts) and the enrichment (FC: fold change) in the three species. (XLSX 123 kb)
Additional file 6: Figure S2. Identification of genes with evolutionary conserved and enriched expression in pancreatic exocrine cells. Venn diagram showing the number of exocrine-enriched genes found only in zebrafish, mouse or human, and those displaying conserved endocrine-enrichment in two species or in the three species (shown in intersections). Due to gene duplications in some species and often in zebrafish, the number of corresponding murine (M) or human (H) orthologous genes is given in brackets in each intersection. The full list of conserved exocrine-enriched genes is given in Additional file 7: Table S5. (PDF 4 kb)
Additional file 7: Table S5. Genes presenting conserved exocrine expression. The ZMH excel page shows the genes with exocrine-enriched expression in zebrafish, mice and human. The ZM, ZH and HM excel pages show genes with endocrine-enriched expression in two species (ZM: zebrafish and mice; ZH: zebrafish and human; HM: human and mice). The tables give the expression mean in endocrine cells (Normalized counts) and the enrichment (FC: fold change) in the three species. (XLSX 101 kb)
Additional file 8: Table S6. List of endocrine-enriched genes with an expression level above 100 counts in embryonic endocrine cells at 27 hpf. List of 911 genes with endocrine-enriched expression in adult and showing an expression level above 100 Normalized counts. Gene expression is provided for the three replicates (selection of pancreatic cells at 27 hpf from Tg(pax6:GFP)) as well as the mean expression level. (XLSX 99 kb)
Additional file 9: Table S7. Classification of endocrine-enriched genes according to their expression in alpha, beta, and delta endocrine cells. Gene expression ratio was calculated for all pair-wise comparison (alpha versus beta: A&B; beta versus delta: B&D and alpha versus delta: A&D). The enrichment is given as Log2 fold change with the adjust value. Classification was done using the cut-off threshold of Log2 fold change > 2 and adjusted *P* < 0.05. (XLSX 265 kb)
Additional file 10: Figure S3. Gene ontology (GO) enrichment analysis for endocrine cell subtypes. Left. Bar plot displaying the number of genes constituting enriched GO terms. *P* values are denoted on the bars. Right. Fold of change (in Log2) of genes constituting the most enriched GO terms, (A) GO enrichment for the 70 beta-enriched genes. (B) GO enrichment for the 73 alpha-enriched genes. (C) GO enrichment for the 192 delta-enriched genes. (B-A: pairwise beta versus alpha; B-D: pairwise beta versus delta). (TIF 13089 kb)
Additional file 11: Figure S4. Expression of *spon1b* gene in beta pancreatic cells of zebrafish embryos. Co-labeling by FISH of *spon1b* with insulin (*ins*) (A, arrows show colocalization), while no expression was detected in delta cells (B) (n > 10). *ins*: insulin, *sst2*: somatostatin 2, N-orth: Endocrine enriched zebrafish gene with no described ortholog in human and/or mouse. (TIF 5116 kb)
Additional file 12: Figure S5. Expression of *pnoca* and *scinlb* genes in alpha pancreatic cells of zebrafish embryos. Co-labeling by in situ hybridization at 30 hpf of new discovered genes with cell type-specific markers for alpha and beta cells (n > 10). A and B. *pnoca* is expressed in alpha cells (A, arrows) while no expression was detected in beta cells (B). *scinlb* was detected specifically in alpha cells (C, arrows) but not detected in beta cells (D). gcga: glucagon a, ins: insulin, Z: no endocrine gene with no conserved expression, N-orth: Endocrine-enriched zebrafish gene with no described ortholog in human and/or mouse. (TIF 8027 kb)
Additional file 13: Figure S6. Validation of the selective expression of some genes in zebrafish delta cells. *cdx1b*, *cdx4*, *lamc2*, and *map3k15* are specifically expressed in delta cells at 24 hpf (A, C, E, G, arrows show colocalization with delta cell-specific markers, somatostatin 2; n > 10). No expression was detected for none of the genes in beta cells (B, D, F, H). *ins*: *insulin*, *sst2: somatostatin 2*, ZHM: Gene expression conserved in zebrafish, human and mouse, ZM: Gene expression conserved in zebrafish and mouse, Z: Endocrine gene with no conserved expression, N-orth: Endocrine-enriched zebrafish gene with no described ortholog in human and/or mouse. (TIF 12250 kb)
Additional file 14: Table S8. Conserved beta cell markers. The ZMH excel page shows the genes with beta cell-enriched expression in zebrafish, mice and human. The ZM, ZH, and HM excel pages show genes with beta cell-enriched expression in two species (ZM: zebrafish and mice; ZH: zebrafish and human; HM: human and mice). Beta cell versus alpha cell expression ratio is given as fold change (FC) for each species. The ZMH, ZH, and HM genes also detected as enriched in specific pancreatic cell types in the human single-cell transcriptomic studies [19–21] are also noted in the columns on the right part. (XLSX 18 kb)
Additional file 15: Table S9. Conserved alpha cell markers. The ZMH excel page shows the genes with alpha cell enriched expression in Zebrafish, Mice and Human. The ZM, ZH and HM excel pages show genes with alpha cell-enriched expression in two species (ZM: Zebrafish and Mice; ZH: Zebrafish and Human; HM: Human and Mice). Alpha cell versus beta cell expression ratio is given as fold change (FC) for the three species. The ZMH, ZH and HM genes also detected as enriched in specific pancreatic cell types in the human single cell transcriptomic studies [19–21] are also noted in the columns on the right part. (XLSX 35 kb)
Additional file 16: Figure S7. Expression and function of myt1b in zebrafish pancreas. A: Whole-mount ISH showing expression pattern of *myt1b* in zebrafish embryos at 27 hpf. High expression is detected in the dorsal pancreatic bud (indicated by the arrow) and in the central nervous system. B: Quantification of glucagon and insulin expression at 48 hpf in wild-type (non-injected) embryos and in embryos injected with the 4 CRISPR *myt1a/b* guide RNA and Cas9. Graph B shows the volume of all gcga^+^ cells and all ins^+^ cells measured in each embryo by the imaging software Imaris (see Methods) (each point is the volume measured in one embryo). This quantification indicates a statistically significant reduction of the volume of alpha cell mass while beta cell mass is not drastically affected in the injected (F0) embryos (results of one experiment). (TIF 2416 kb)
Additional file 17: Table S10. Primers used for RNA probe synthesis. List of primers used for the synthesis of the RNA probes for in situ hybridization, antisense primers contain the T3 minimal promoter sequence. (XLSX 10 kb)

## Abbreviations

dpf: Days post fertilization
FISH: Fluorescent in situ hybridization
gcga: Glucagon a
GPCRs: G-couple protein receptors
hpf: Hours post fertilization
ins: Insulin
PCA: Principal component analysis
sst2: Somatostatin 2
WISH: Whole-mount in situ hybridization
ZH: Genes with conserved expression pattern in zebrafish and human
ZM: Genes with conserved expression pattern in zebrafish and mice
ZMH: Genes with conserved expression pattern in zebrafish, mice and human
