# Supplementary figures and images for: Transcriptome analysis of pancreatic cells across distant species highlights novel important regulator genes
Source: BMC Biol. 2017 Mar 21;15:21. doi: 10.1186/s12915-017-0362-x (PMC5360028; doi:10.1186/s12915-017-0362-x)

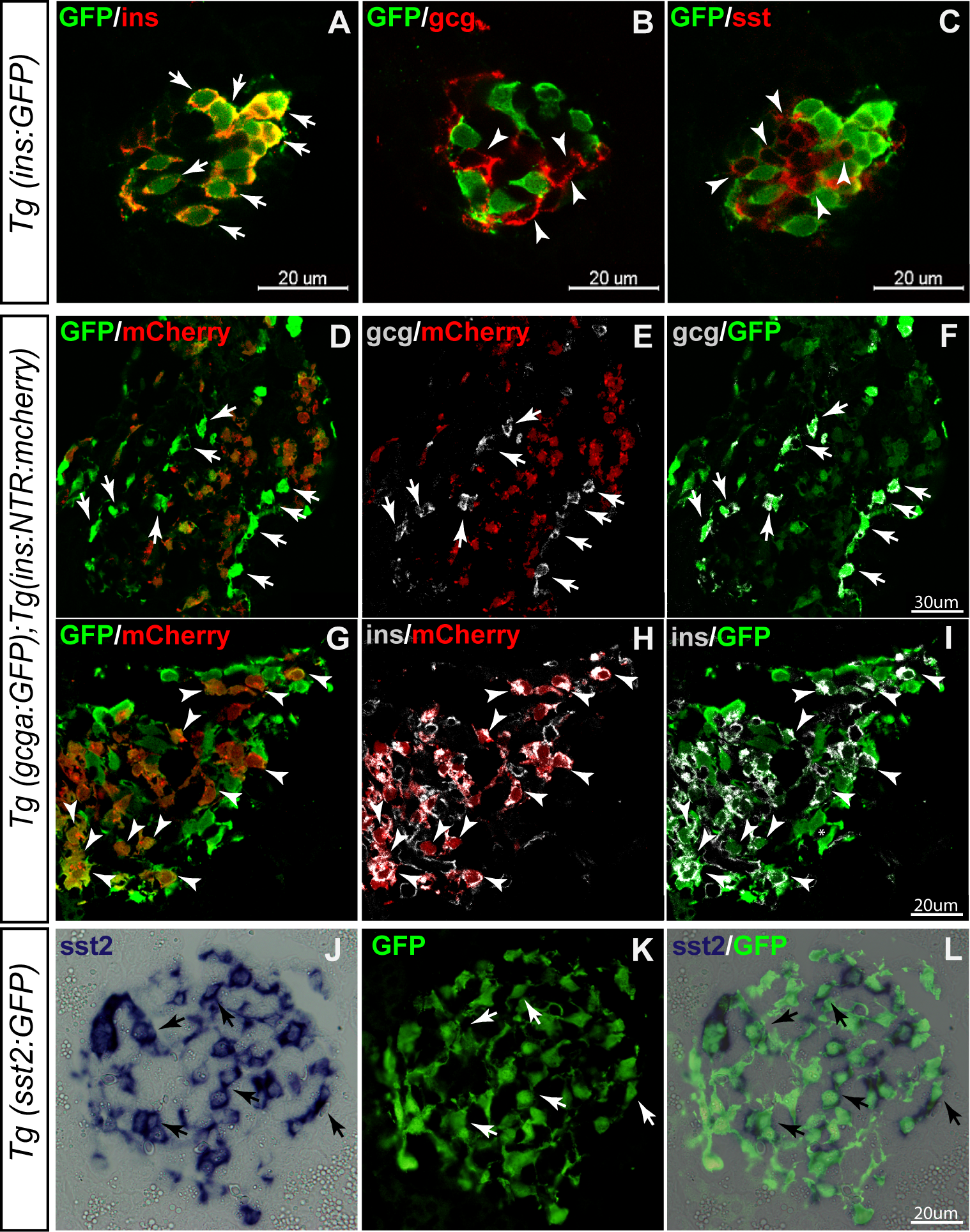

Supplement: Additional file 1: Figure S1. — Expression of the transgenes Tg(ins:GFP), Tg(gcga:GFP), Tg(sst2:GFP), and Tg(ins:NTR:mCherry) in the endocrine pancreatic cell types. (A–C) Whole mount immunostaining of 3 dpf transgenic larvae Tg(ins:GFP) demonstrating the selective expression of GFP in beta cells. (D–I) Immunostaining of pancreas sections from adult transgenic fish Tg(gcga:GFP/ins:NTR:mCherry). Glucagon+ cells (see arrows in D–F) express high level of GFP and are not labelled by mCherry, while many insulin expressing cells are labelled by mCherry and by GFP (at slightly lower levels) (see arrowheads in G–I). These data reveal a leaking expression of the gcga:GFP transgene in beta cells. (J–L) ISH performed on pancreas section of adult Tg(sst2:gfp) with sst2 probe followed by immunofluorescence using the GFP antibody; the expression of endogenous sst2 gene co-localize with GFP staining confirming the specific expression of the transgene in delta cells [28]. (TIF 8638 kb) [file 12915_2017_362_MOESM1_ESM.tif]

# Exocrine

Zebrafish

Human

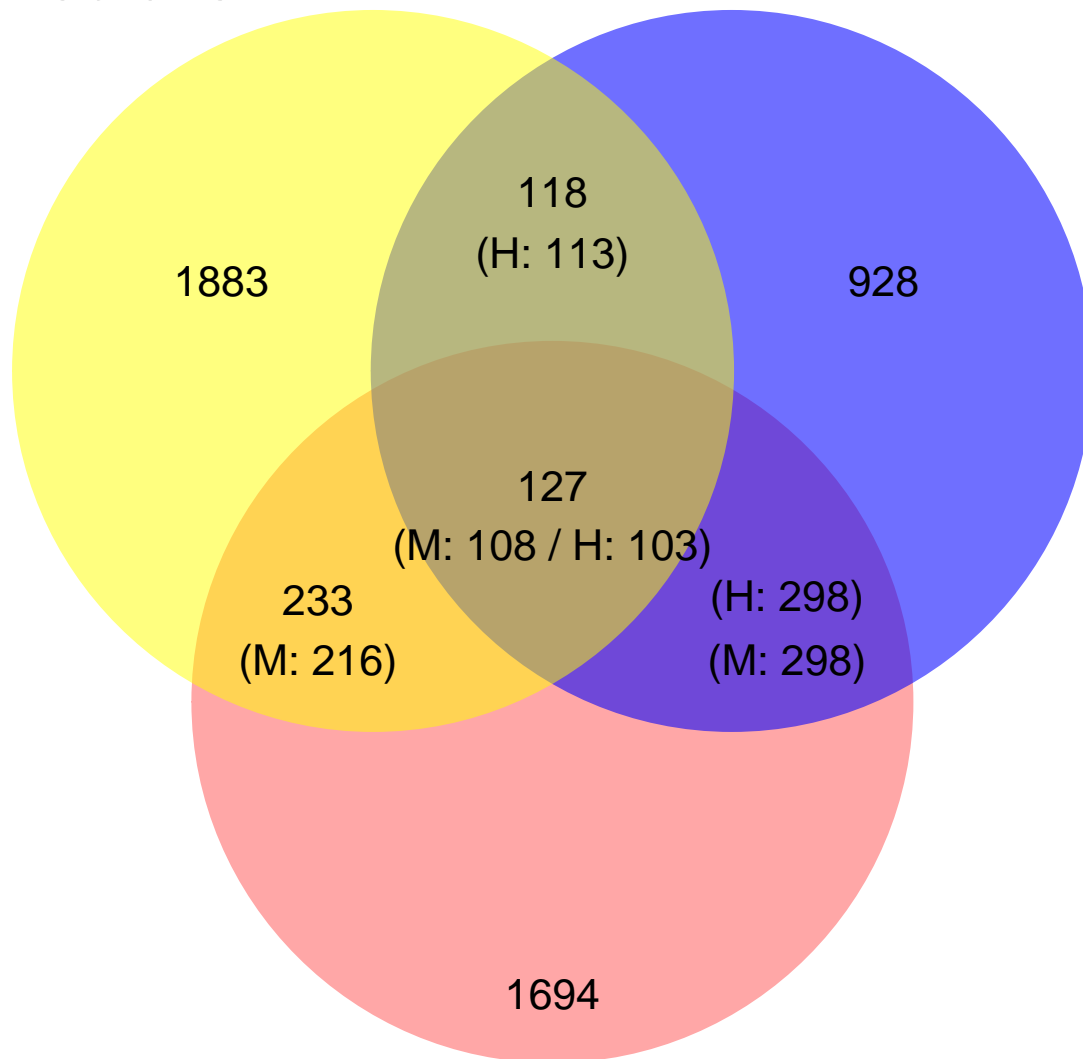

Mouse

Supplement: Additional file 6: Figure S2. — Identification of genes with evolutionary conserved and enriched expression in pancreatic exocrine cells. Venn diagram showing the number of exocrine-enriched genes found only in zebrafish, mouse or human, and those displaying conserved endocrine-enrichment in two species or in the three species (shown in intersections). Due to gene duplications in some species and often in zebrafish, the number of corresponding murine (M) or human (H) orthologous genes is given in brackets in each intersection. The full list of conserved exocrine-enriched genes is given in Additional file 7: Table S5. (PDF 4 kb) [file 12915_2017_362_MOESM6_ESM.pdf]

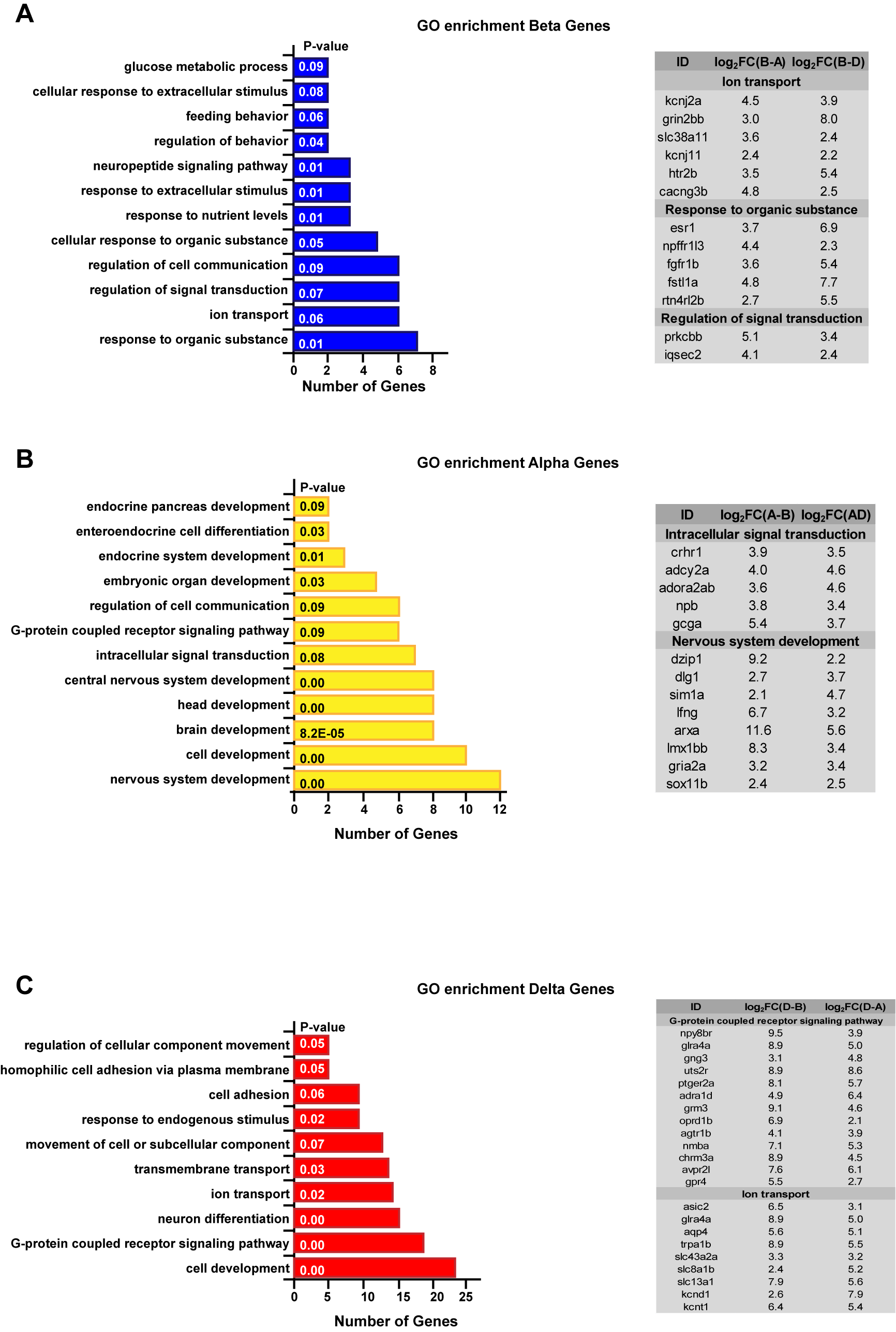

Supplement: Additional file 10: Figure S3. — Gene ontology (GO) enrichment analysis for endocrine cell subtypes. Left. Bar plot displaying the number of genes constituting enriched GO terms. P values are denoted on the bars. Right. Fold of change (in Log2) of genes constituting the most enriched GO terms, (A) GO enrichment for the 70 beta-enriched genes. (B) GO enrichment for the 73 alpha-enriched genes. (C) GO enrichment for the 192 delta-enriched genes. (B-A: pairwise beta versus alpha; B-D: pairwise beta versus delta). (TIF 13089 kb) [file 12915_2017_362_MOESM10_ESM.tif]

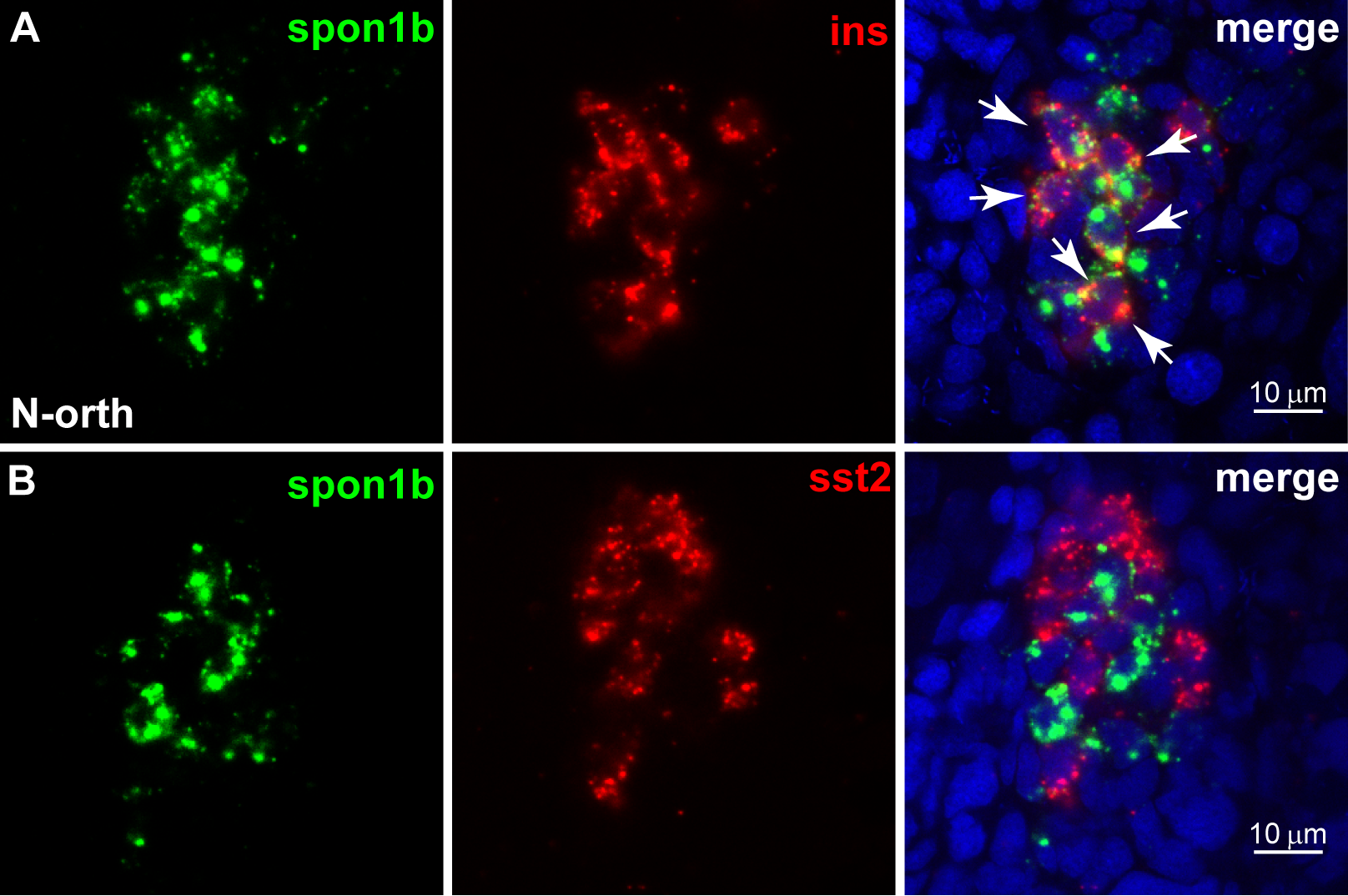

Supplement: Additional file 11: Figure S4. — Expression of spon1b gene in beta pancreatic cells of zebrafish embryos. Co-labeling by FISH of spon1b with insulin (ins) (A, arrows show colocalization), while no expression was detected in delta cells (B) (n > 10). ins: insulin, sst2: somatostatin 2, N-orth: Endocrine enriched zebrafish gene with no described ortholog in human and/or mouse. (TIF 5116 kb) [file 12915_2017_362_MOESM11_ESM.tif]

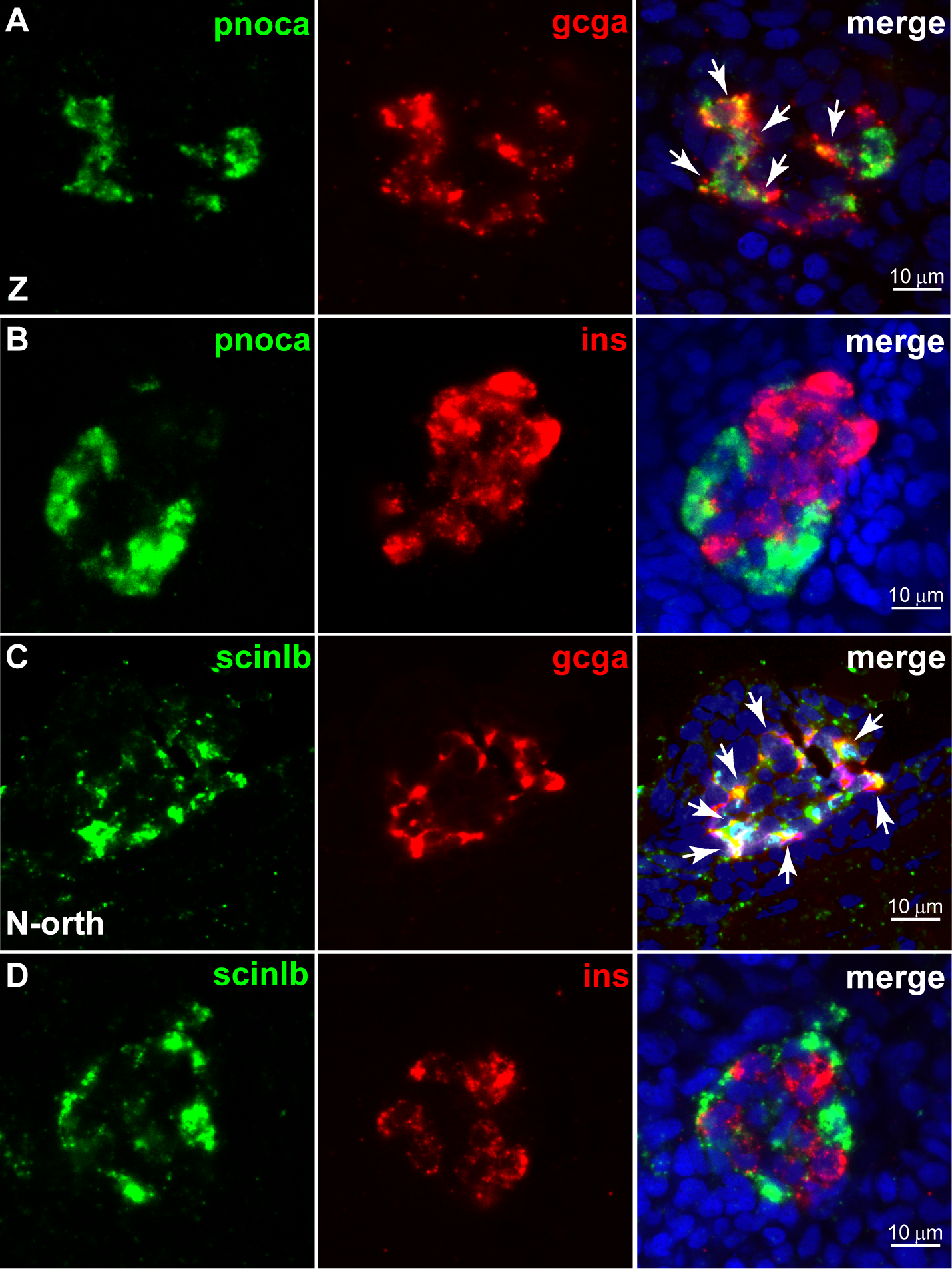

Supplement: Additional file 12: Figure S5. — Expression of pnoca and scinlb genes in alpha pancreatic cells of zebrafish embryos. Co-labeling by in situ hybridization at 30 hpf of new discovered genes with cell type-specific markers for alpha and beta cells (n > 10). A and B. pnoca is expressed in alpha cells (A, arrows) while no expression was detected in beta cells (B). scinlb was detected specifically in alpha cells (C, arrows) but not detected in beta cells (D). gcga: glucagon a, ins: insulin, Z: no endocrine gene with no conserved expression, N-orth: Endocrine-enriched zebrafish gene with no described ortholog in human and/or mouse. (TIF 8027 kb) [file 12915_2017_362_MOESM12_ESM.tif]

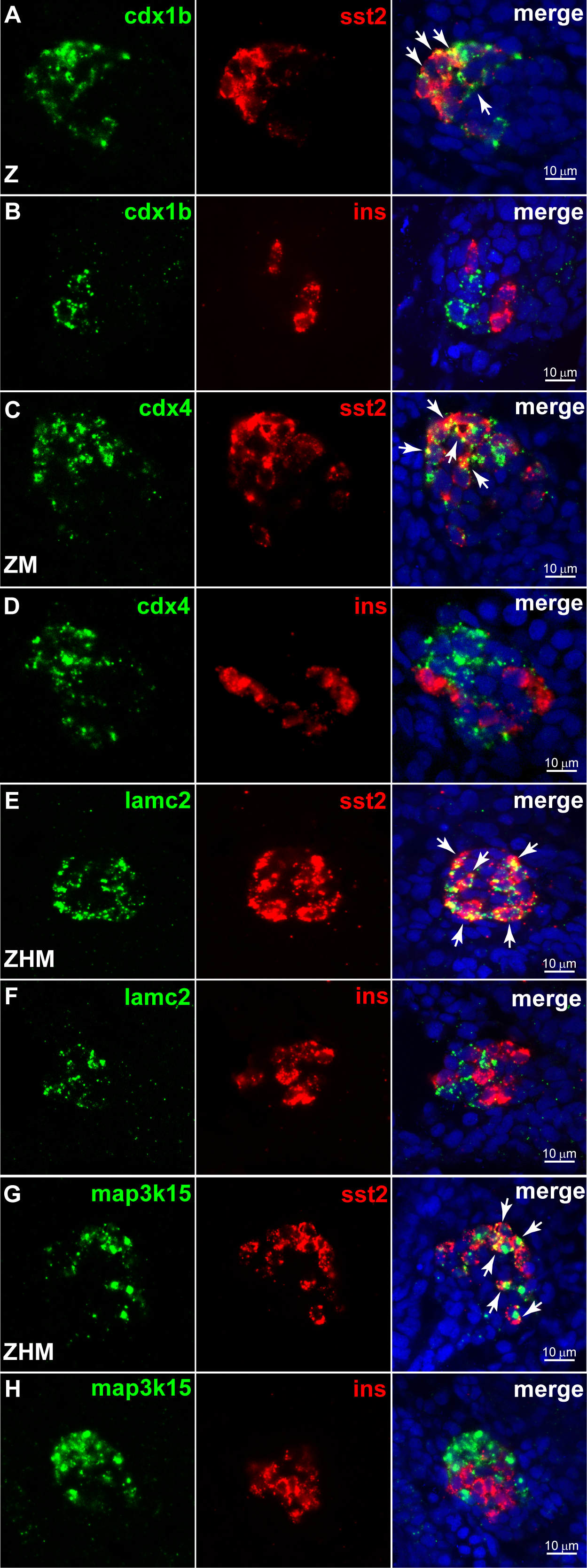

Supplement: Additional file 13: Figure S6. — Validation of the selective expression of some genes in zebrafish delta cells. cdx1b, cdx4, lamc2, and map3k15 are specifically expressed in delta cells at 24 hpf (A, C, E, G, arrows show colocalization with delta cell-specific markers, somatostatin 2; n > 10). No expression was detected for none of the genes in beta cells (B, D, F, H). ins: insulin, sst2: somatostatin 2, ZHM: Gene expression conserved in zebrafish, human and mouse, ZM: Gene expression conserved in zebrafish and mouse, Z: Endocrine gene with no conserved expression, N-orth: Endocrine-enriched zebrafish gene with no described ortholog in human and/or mouse. (TIF 12250 kb) [file 12915_2017_362_MOESM13_ESM.tif]

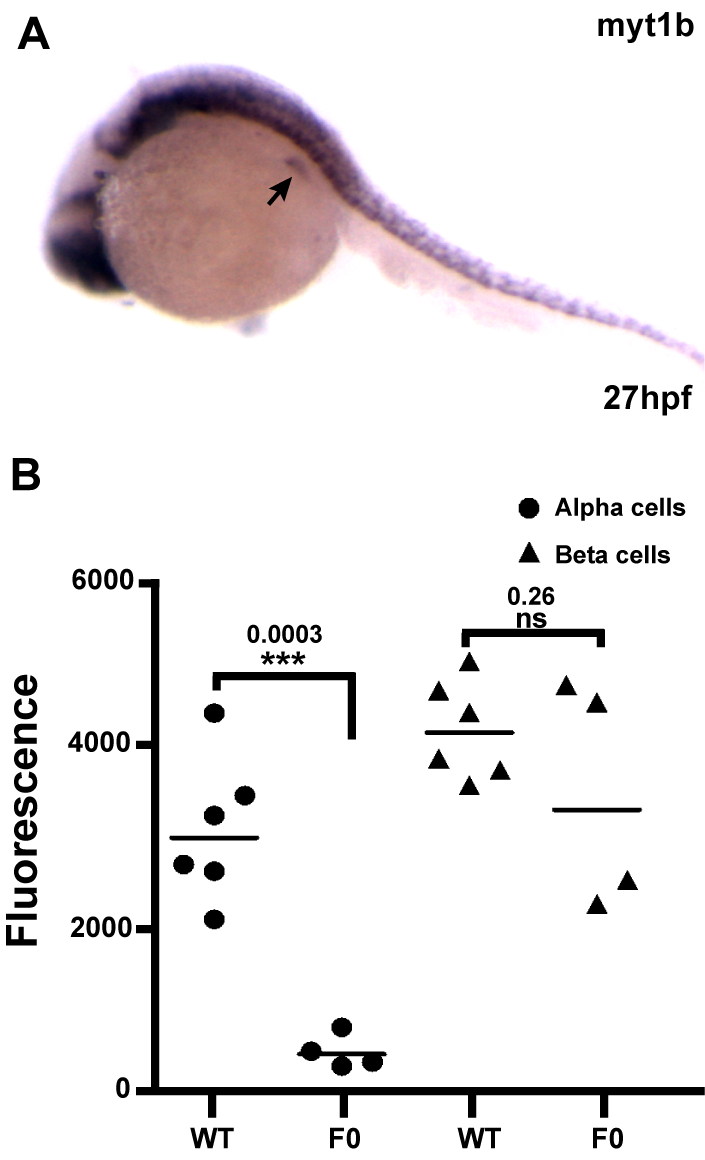

Supplement: Additional file 16: Figure S7. — Expression and function of myt1b in zebrafish pancreas. A: Whole-mount ISH showing expression pattern of myt1b in zebrafish embryos at 27 hpf. High expression is detected in the dorsal pancreatic bud (indicated by the arrow) and in the central nervous system. B: Quantification of glucagon and insulin expression at 48 hpf in wild-type (non-injected) embryos and in embryos injected with the 4 CRISPR myt1a/b guide RNA and Cas9. Graph B shows the volume of all gcga+ cells and all ins+ cells measured in each embryo by the imaging software Imaris (see Methods) (each point is the volume measured in one embryo). This quantification indicates a statistically significant reduction of the volume of alpha cell mass while beta cell mass is not drastically affected in the injected (F0) embryos (results of one experiment). (TIF 2416 kb) [file 12915_2017_362_MOESM16_ESM.tif]
